# Live birth in patients stimulated with r-hFSH or r-hFSH: r-hLH is strongly associated with cumulus cell derived gene expression models

**DOI:** 10.1186/s12958-025-01480-2

**Published:** 2025-11-22

**Authors:** T. Adriaenssens, I. Van Vaerenbergh, W. Van Leuven, W. Coucke, S. Montenegro, W. Zheng, T. D’Hooghe, L. Van Landuyt, N. De Munck, E. Van Hecke, J. Smitz, A. Rosenthal, C. Blockeel

**Affiliations:** 1 Fertiga, Brussels, Belgium; 2 Sciensano, Quality of Laboratories, Brussels, Belgium; 3 Global Medical Affairs Fertility, Merck KGaA, Darmstadt, Germany; 4 UZ Brussel, Brussels IVF, and Genetics Reproduction and Development (GRAD), Vrije Universiteit Brussel, Brussels, Belgium

**Keywords:** Cumulus cells, Gene expression, Recombinant gonadotropins, Ovarian stimulation, Oocyte competence

## Abstract

**Background:**

Morphological embryo assessment, time-lapse imaging or PGT-A can prioritize an embryo for transfer in IVF/ICSI cycles. Nevertheless, there remains potential to enhance the efficiency of ART cycles and reduce the time-to-pregnancy. Previously, a pregnancy predictive non-invasive cumulus cell (CC) test was developed and clinically validated for HP-hMG stimulated patients. In this study, CC gene expression profiles from r-hFSH and r-hFSH:r-hLH stimulated ICSI patients were evaluated for their potential to predict the most competent oocyte/embryo, resulting in live birth.

**Methods:**

This observational cohort study comprises 113 patients allocated to the two study groups stimulated with either r-hFSH (*n* = 47) or r-hFSH:r-hLH (*n* = 66). RT-qPCR analysis was performed on 1135 CC samples for 11 predefined biomarkers (CAMK1D, EFNB2, SASH1, GOT1, SLC6A9, HAS2, PTGS2, HSPH1, VCAN, GSTA4, STC2) and 2 endogenous controls (UBC, B2M). Univariate (95%CI) and multivariable analyses (leave-one-out cross-validation and stepwise linear regression) were performed.

**Results:**

The two study groups were first compared to verify if one prediction model could fit the two patient groups. While patient characteristics and stimulation were comparable, the biomarker expression for EFNB2 (e.g.: CC of all oocytes *n* = 1123, 95%CI: 0.27, 0.49) and GOT1 (CC of all oocytes *n* = 1123, 95%CI: -0.25, -0.11) significantly differed between the two groups.

Stepwise linear regression models were therefore built for the two study groups. The biomarker expression in CC of oocytes developing into transferred blastocysts was compared based on transfer outcomes (live birth or not) and models contained only gene expression data. The strongest live birth predictive biomarkers were GOT1, HAS2, SASH1 and PTGS2 for r-hFSH patients (AUC 0.7284; 70% accuracy) and GOT1 with HAS2 (AUC 0.9529; 88% accuracy) for r-hFSH:r-hLH stimulated patients.

**Conclusions:**

These findings merit further validation in an interventional prospective study. Predictive CC biomarkers are a promising non-invasive technology to shorten the time-to-pregnancy in ICSI patients stimulated with different types of recombinant gonadotropins.

**Trial registration:**

Ethical approval was obtained from the Ethical Committee of Vrije Universiteit Brussel – UZ Brussel (IEC: 2020.335) and the study was registered at ClinicalTrials.gov (ID NCT04710264; registration date 14/1/2021).

**Supplementary Information:**

The online version contains supplementary material available at 10.1186/s12958-025-01480-2.

## Background

For the past four decades, assisted reproductive technologies (ART), including ovarian stimulation combined with in-vitro fertilization (IVF) or intracytoplasmic sperm injection (ICSI), have been utilized to address fertility challenges. These procedures involve single embryo transfer (SET)—either fresh or frozen—or double embryo transfer (DET) on day 3, or SET on day 5, to enhance conception rates in infertile couples.

The decision to transfer a specific embryo typically relies on morphological assessment of the embryos. Until today, traditional morphological embryo scoring is still considered the gold standard for ranking embryos for transfer.

Invasive embryo biopsy techniques, such as Preimplantation Genetic Testing for aneuploidy (PGT-A), are employed to detect chromosomal abnormalities typically on a subset of blastocyst cells and to inform decisions regarding embryo transfer. However, the utility of PGT-A as a prognostic screening tool remains under debate [1]. In a randomized clinical trial, the benefit of PGT-A on blastocysts was only demonstrated for patients over 35 years of age [2], a finding further supported by a meta-analysis [3]. Concerns regarding the reliability of invasive embryo selection methods, such as trophectoderm biopsy in PGT-A, have also been raised with evidence suggesting that viable embryos may be unnecessarily excluded [4]. By showing that euploid embryos have a high likelihood of implantation across multiple attempts, the study highlighted the limitations of relying solely on invasive selection techniques and emphasized the need for careful interpretation of PGT-A results. These observations underscore the importance of continued research into alternative, less invasive embryo selection strategies. One such approach, non-invasive PGT-A on spent blastocyst media, has been proposed; however, its reported sensitivity and specificity remain limited [5], reviewed by [6].

Time-lapse imaging and artificial intelligence (AI) have been introduced more recently to the embryology lab to optimize the embryo ranking and selection for transfer (review by [7]. The goal is to reduce the time-to-pregnancy leading to a healthy live birth. However, a Cochrane review on time-lapse systems showed low-quality evidence that time-lapsed imaging increased live birth and clinical pregnancy in the first transfer [8]. Furthermore, unlike time-lapse imaging, which has been studied both retrospectively and prospectively (including RCTs) over the past decade, AI-based embryo selection is still in the exploratory stage (review by [9]).

Nonetheless, there is still a large need to improve clinical outcomes and shorten the time-to-pregnancy in ICSI cycles, ideally through the application of other non-invasive methods. mRNA expression profiles in cumulus cells have been identified as indicators of oocyte and embryo developmental potential in ART treatments [10, 11]. Oocyte developmental competence is defined as the capacity of the oocyte to be fertilized, sustain development to the blastocyst stage and subsequently lead to implantation, clinical pregnancy and/or live birth [12].

There are multiple advantages to cumulus cell (CC) based oocyte/embryo grading: 1) CC are somatic cells that surround and support the oocyte during its growth and maturation and are as such an excellent surrogate cell source for correlation analysis [10, 13]. 2) During an ICSI treatment, CC are removed from the oocyte before injection as part of standard care and are considered ‘waste material’ from the procedure, as such avoiding certain legal and ethical concerns that may be relevant for PGT-A blastocyst biopsies. 3) The test can be performed within one day, and allows transfer in the fresh pick up cycle, while PGT-A requires vitrification of the blastocysts and will delay the embryo transfer. 4) This test may also add critical information on oocyte competence for women considering oocyte freezing for fertility preservation or oocyte donation cycles. 5) Alternatively, the test can be used to identify the most competent oocytes/embryos and limit the number of oocytes to be fertilised/grown and thus limit the number of supernumerary embryos generated (e.g. embryo protection law Germany [14]. It also could limit the number of embryos needed to be biopsied for PGT-A. 6) Most importantly, the test reduces the stress and burden of failed transfers for the patient by significantly improving the live birth rate per transfer, and shortens time-to-pregnancy.

The main disadvantage of the test is that it requires extra hands-on time for the individual denudation of the oocytes, but this is limited and all alternative technologies also require extra expertise and hands-on time for biopsies, extra washing of COC/blastocysts, or extended picture/pattern observations. A minor remark is that the test is currently only applied on ICSI patients. This is to ascertain sample homogeneity and avoid sample drop out due to too few cells, so only for technical reasons.

Previously, a non-invasive cumulus cell test predictive of pregnancy was developed and clinically validated by our group for HP-hMG stimulated ICSI patients [15, 16]. However, it has been established by our group [17] and confirmed by others [18, 19] that the hormone class used for ovarian stimulation influences the cumulus cell gene expression. Other groups have retrospectively studied numerous cumulus cell predictive biomarkers (reviewed by [20]). Until now, a similar predictive test for r-FSH stimulated patients has been lacking.

For the discovery of r-FSH specific tests, cumulus cells from patients stimulated with either r-hFSH (Gonal-*f*, follitropin alfa) or r-hFSH: r-hLH (Pergoveris, combination of follitropin alfa and lutropin alfa in a 2:1 ratio) were analysed using gene expression profiles of selected biomarker genes by RT-qPCR. These biomarkers were selected from our patent portfolio of 45 differentially expressed genes from prior discovery work. This prior work involved high throughput screening of independent patients using Affymetrix microarrays, with validation conducted through qPCR (not published).

The aim of the current study was to identify CC gene expression profiles predictive for live birth in ICSI patients stimulated with either r-hFSH or r-hFSH: r-hLH. This was achieved by comparing the expression of key biomarkers between both patient groups to determine whether a single predictive model could be applied to both or if separate models would be necessary. Subsequently, cross-validation was employed to identify the most important biomarkers from a set of 11 biomarker genes, and stepwise linear regression was used to construct models for live birth outcomes.

## Methods

### Patient inclusion/exclusion criteria

This observational cohort study was conducted at the Universitair Ziekenhuis Brussel (Brussels IVF, Brussels, Belgium) from January 2021 until June 2023. Ethical approval was obtained from the Ethical Committee of Vrije Universiteit Brussel – UZ Brussel (IEC: 2020.335) and the study was registered at ClinicalTrials.gov (ID NCT04710264).

Patients aged 22–38 years, with a BMI between 17–33 kg/m2, were included in the study upon providing written informed consent. These patients underwent their first or second ICSI treatment cycle, and consented to individual oocyte denudation, RNA expression testing using their cumulus cells, and a fresh single blastocyst transfer. Eligibility required patients to be good ovarian responders defined as having an AMH level between 1 to 4.7 ng/ml and 7 to 18 follicles measuring 10–11 mm on the last ultrasound. Patients agreed to receive either r-hFSH (Gonal-*f*) or r-hFSH: r-hLH (Pergoveris) using a GnRH antagonist protocol with hCG trigger.

Women with a history of low oocyte maturation or known maturation defect, irregular menstrual cycles (< 24 or > 35 days), BMI < 17 or > 33, smoking > 10 cigarettes per day, known low ovarian response based on the Bologna criteria [21], PCOS (polycystic ovary syndrome) defined by revised criteria ASRM ESHRE 2018 [22], severe endometriosis ≥ III (AFS classification), or combined use of urinary and recombinant gonadotropins in the current ovarian stimulation cycle were excluded from the study. Additionally, patients scheduled for PGT (preimplantation genetic testing), TESE (testicular sperm extraction) or couples with extreme oligo-astheno-teratozoospermia (OAT) with a sperm count below 100,000/ml were also excluded. Study participants were not enrolled in any other concurrent studies.

### Sample size

The sample size calculation was based on three assumptions: (i) an ongoing pregnancy rate (OPR) of approximately 50% for fresh blastocyst transfers at UZ Brussels (ii) significant differences in gene expression between the two patient groups, and (iii) the need for at least 20 patients with positive OPR in each group to develop predictive gene expression models for OPR based on previous experiments [23]. Consequently, 40 patients for each group were initially planned, totalling 80 patients. An interim analysis was conducted as planned after 40 patients had been recruited. Due to an observed lower than expected OPR in both groups, recruitment continued until each group had at least 20 patients with positive OPR outcome.

### Objectives

The primary objective was to identify potential differences in the expression of five key biomarkers genes in the CC of the two patient groups and to determine whether a unified prediction model could be applied or if separate models were necessary. The secondary objective was to evaluate the expression of 11 biomarkers for their potential to predict live birth outcomes utilizing cross-validation and linear regression modelling techniques.

### Ovarian stimulation, ICSI, embryo culture, cumulus cells collection and outcomes

Ovarian stimulation was conducted using a GnRH antagonist protocol, with patients receiving either r-hFSH (Gonal-*f*, follitropin alfa) or r-hFSH: r-hLH (Pergoveris, combination of follitropin alfa and lutropin alfa in a 2:1 ratio) at a starting dose of r-hFSH ranging between 150–225 IU. Final oocyte maturation was induced by administering 250 μg of r-hCG (Ovitrelle) when at least 3 follicles ≥ 17 mm were observed by transvaginal ultrasound. Oocyte retrieval (OR) was scheduled 36 h post-trigger. Luteal phase support was provided using micronized vaginal progesterone, initiated according to the centres’ standard of care, and continued until ultrasound confirmation of a foetal sac with a heartbeat at 7 weeks of gestation.

Individual denudation was performed on day 0 by enzymatic (Cumulase, Origio, CooperSurgical) and mechanical removal of cumulus cells [24] within two hours after oocyte retrieval. After individual denudation of all cumulus oocyte complexes (COC), ICSI was performed on all mature oocytes. Sperm motility was assessed according to the Tygerberg criteria [25]. Mature oocytes were in the second phase of meiosis (metaphase II or MII). Oocytes with two visible pronuclei (2PN) were considered normally fertilised. The collection of cumulus cells has been described before [16]. All individual cumulus cells were collected and snap-frozen in liquid nitrogen. Cumulus cell samples of all oocytes were transported at −196 °C to the molecular laboratory of Fertiga, Belgium and stored at −80 °C for further molecular analysis.

Embryo culture procedures were previously described [26]. Briefly, ten individual 25μl media droplets were placed in a circular arrangement in an embryo culture dish (IVF round dish, Ø 60 mm, 353801, Falcon) with four additional 25μl media droplets positioned centrally. All droplets were then covered with 7 ml Ovoil (Vitrolife, Sweden). Following preparation, embryo culture dishes were equilibrated overnight in a tri-gas incubator containing 5% O2, 6% CO2, and 89% N2 at 37 °C. Single embryo culture was carried out from day 0 post-ICSI until day 3 in cleavage medium (OS Cleav, 83040010 A, Origio, Denmark). On day 3, embryos were transferred to blastocyst medium (OS Blast, 83060010 A, Origio, Denmark) and cultured until day 5 or 6.

Embryo quality was scored using established grading systems. On day 3, cleavage-stage embryos were assessed and categorized as excellent, good, moderate or poor [27]. On day 5 blastocysts were categorized in the same four groups [28]. On day 5, all blastocysts were scored according to Gardner and Schoolcraft [29]. Good quality embryos in the current study are the embryos considered “good” and “fair” according to the ESHRE guidelines [30]. A single fresh blastocyst was selected for transfer based on these morphological criteria. Supernumerary blastocysts were vitrified individually using CBS high security straws in combination with VitNX (Fujifilm) for use in subsequent frozen embryo transfer cycles [31].

A βhCG positive pregnancy was defined as the detection of βhCG in the serum, indicating early pregnancy. Clinical pregnancy was defined as a pregnancy diagnosed by ultrasonographic or clinical documentation of at least one foetus with a discernible heartbeat in gestational week 6 to 8. An ongoing pregnancy was defined as a pregnancy defined by the presence of a gestational sac and a detectable foetal heart beat assessed by transvaginal ultrasound between 10 to 12 weeks of gestation. Live birth was defined as the birth of at least one newborn after 24 weeks of gestation who exhibits any sign of life.

### RNA extraction and RT-qPCR

The CC RNA analysis was conducted at the Fertiga laboratory following the inclusion of all patients in the study. Total RNA was extracted using the RNeasy Micro kit (Qiagen, The Netherlands) with the Qiacube Connect (Qiagen). Subsequently, RNA was reverse transcribed into complementary DNA (cDNA) using the iScript cDNA synthesis kit (BioRad, Belgium) following the manufacturer’s protocol. The cDNA was stored at − 80 °C until further quantitative PCR analysis.

Quantitative PCR (qPCR) was performed in triplicate for the following 11 biomarkers: CAMK1D (Calcium/Calmodulin Dependent Protein Kinase ID), EFNB2 (Ephrin B2), SASH1 (SAM And SH3 Domain Containing 1), GOT1 (Glutamic-Oxaloacetic Transaminase 1), SLC6A9 (Solute Carrier Family 6 Member 9), STC2 (Stanniocalcin 2), HAS2 (Hyaluronan Synthase 2), VCAN (Versican), PTGS2 (Prostaglandin-Endoperoxide Synthase 2), HSPH1 (Heat Shock Protein Family H (Hsp110) Member 1), GSTA4 (Glutathione S-Transferase Alpha 4), and for two endogenous control genes: B2M (Beta-2-Microglobulin) and UBC (Ubiquitin C). These genes were selected from our portfolio of patented genes and were previously identified by our group using microarrays in r-hFSH and HP-hMG stimulated patients. Some of these genes were already validated with qPCR in previous published studies [15, 23, 32, 33].

The qPCR analysis was done in two steps. First, CC from all 1,123 individually denudated oocytes were analysed for five marker genes (CAMK1D, EFNB2, SASH1, GOT1, SLC6A9) and two endogenous control genes (B2M, UBC). Subsequently, CC samples from 341 selected oocytes were analysed for the following six additional marker genes (STC2, HAS2, VCAN, PTGS2, HSPH1, GSTA4). Specificity of amplification during qPCR was assessed using melting curve analysis. Relative quantification of gene expression was performed using standard curves generated from serial dilutions of synthetic oligonucleotides corresponding to each amplicon. Gene expression data were normalized to the mean of the endogenous control genes and log_2_ transformed prior to statistical analysis to approximate a Gaussian distribution, facilitating parametric statistical analysis.

### Analysis strategies and statistical methods used

To detect potential differences between the two patient groups, patient characteristics, embryology, stimulation and cycle outcomes measures were compared with Mann–Whitney tests or two sided Fisher’s exact tests in GraphPad Prism V10. A significance threshold of *p* < 0.05 was considered statistically significant. To correct for multiple testing in the Mann–Whitney analyses, a Bonferroni correction was applied adjusting the significance threshold to *p* < 0.0025.

For the analysis of cumulus cell (CC) gene expression in this study, two distinct cohorts were established based on treatment protocols and oocyte competence.

#### Comprehensive gene expression analysis

A total of 1,123 CC samples were analysed for five key biomarkers: SASH1, EFNB2, CAMK1D, GOT1, SLC6A9. These genes were selected based on their roles in calcium homeostasis, cell signalling, and metabolic process, which are critical for oocyte maturation and embryo development. The analysis aimed to compare gene expression profiles between two treatment protocols: r-hFSH and a combination of r-hFSH with r-hLH.

#### Selective gene expression analysis based on oocyte competence

In the second phase of the study, gene expression in CCs was analysed from a subset of 146 oocytes that resulted in a transferred blastocyst. These CCs were analysed for six additional biomarkers: PTGS2, VCAN, HSPH1, HAS2, GSTA4, and STC2. These genes were chosen for their involvement in prostaglandin synthesis, extracellular matrix remodelling, stress response, and antioxidant defence, all of which are pertinent to oocyte quality and embryo development.

By employing these two analytical strategies, the study aims to elucidate the impact of different gonadotropin treatments on CC gene expression and to identify biomarkers predictive of oocyte competence and IVF success.

### Correlation analysis methodology

To investigate potential differences between the patient groups, correlation analyses were performed between clinical variables, stimulation, outcome measures and gene expression data (average expressions of all MII per patient). Separate analyses were conducted for patients treated with r-hFSH and those treated with r-hFSH: r-hLH as well as for the combined patient cohort. Prior to the correlation analyses, all parameters were assessed for a normal distribution. Log_2_ transformations were applied to gene expression data and via Arc Sin transformations were applied to percentage variables to approximate normal distributions. Normal Q-Q (quantile–quantile) plots of residuals were generated to visually assess the distribution of the data. Despite these transformations not all variables achieved normality. Consequently, Spearman’s rank correlation coefficient r was calculated to evaluate monotonic relationship between variables. A significance threshold was established using the Bonferroni correction to account for multiple comparisons. Given 22 tests, the adjusted significance level was set at *p* < 0.0023. Correlations with |r|> 0.5 and *p* < 0.0023 were considered statistically significant and were further analysed.

### Primary objective analysis methodology

For the primary objective, gene expression data of five key biomarkers (CAMK1D, EFNB2, SASH1, GOT1 and SLC6A9) were analysed across five distinct datasets: (i) CC of all oocytes, (ii) CC of all metaphase II (MII) oocytes, (iii) CC of all normally fertilized (2PN) oocytes, (iv) CC of all fertilized oocytes developing into Good-Quality Embryos (GQE) on day 3, and (v) CC of all fertilized oocytes developing into a GQE on day 5.

One-way parametric analyses were performed in GraphPad Prism V10, calculating 95% confidence intervals (CIs) to assess differences between treatment protocols. These analyses aimed to exclude potential biases related to oocyte competence. Additionally, a multivariate analysis of variance (MANOVA) was conducted in R on the same biomarkers across two datasets: (i) CC of all oocytes (the largest dataset) and (ii) CC of all fertilized oocytes developing into a GQE on day 5 (the most specific dataset).

### Secondary objective analysis methodology

For the secondary objective, gene expression data of 11 biomarkers (CAMK1D, EFNB2, SASH1, GOT1, SLC6A9, HAS2, PTGS2, HSPH1, VCAN, GSTA4, STC2) were analysed to identify predictive biomarkers for patients stimulated with r-hFSH as well as with a combination of r-hFSH and r-hLH. The analysis focused on CC from oocytes that either developed into transferable blastocysts leading to live birth or did not. This analysis was an inter-patient analysis.

### Model development and validation

A “leave-one-out” cross-validation approach was employed to mitigate the risk of overfitting and ensure the robustness of the predictive model. Model construction utilized stepwise regression analysis based on a generalized linear mixed model (GLMM), incorporating patient as a random effect and biomarkers as fixed explanatory variables. Both forward and backward stepwise regression analysis was applied to identify the most predictive biomarkers, resulting in multivariate models containing up to four gene expression variables. This approach has been successfully applied in previous studies to identify predictive RNA profiles in fertility research [15, 16].

### Visualisation of relationships

To facilitate visual interpretation of the relation between gene expression and live birth outcome, scatter plots were generated with gene expression on the X-axis and predicted live birth outcomes on the Y-axis. A Locally Weighted Scatterplot Smoothed (LOWESS) line was included to highlight trends in the data. This visualization approach abstracts from other variables to provide a clear representation of the overall positive or negative relationships between gene expression and live birth outcomes.

## Results

### Study participants flow, drop-out rationale, study patient allocation

**Fig. 1:**
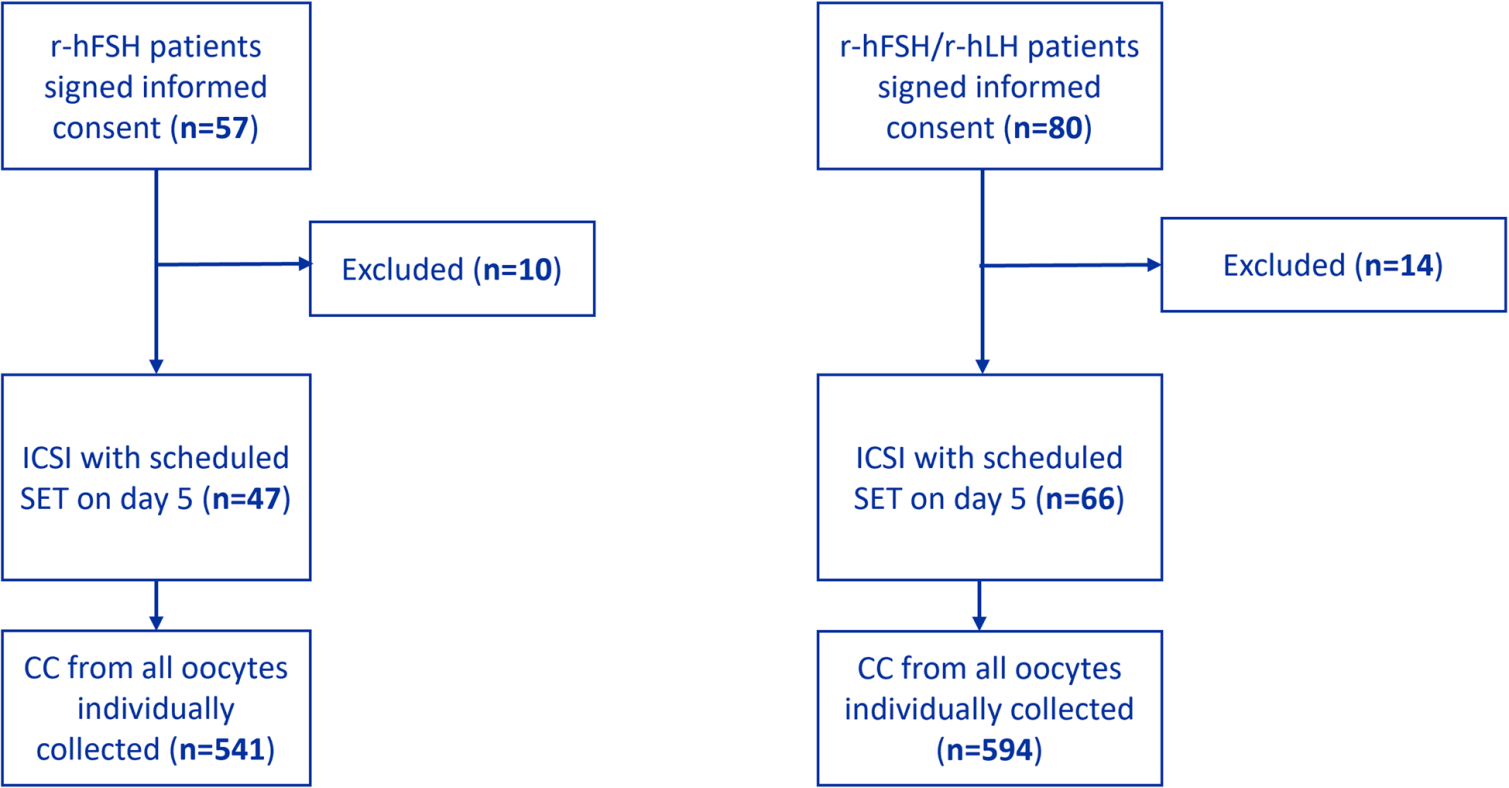
Flowchart patient inclusion and allocation to the two study groups. For all 113 patients, cumulus cells from all oocytes were individually collected on the day of oocyte retrieval, ICSI was performed and SET was scheduled on day 5

In this single centre study, 137 patients were initially recruited and signed the informed consent. Of these, 24 patients (10 in the r-hFSH group and 14 in the r-hFSH: r-hLH group) were considered as drop-outs for the study (Fig. 1) due to the following reasons: (i) Covid infection (*n* = 2), (ii) freeze-all decision at the time of oocyte retrieval (*n* = 13), (iii) low sperm count (*n* = 1), (iv) BMI > 33 kg/m2 (*n* = 1), (v) withdrawn by the investigator (*n* = 6), and (vi) technical reasons (due to an incorrect sample transport) (*n* = 1).

The remaining 113 patients were assigned into one of the two study groups: 47 received stimulation with r-hFSH and 66 patients were treated with r-hFSH: r-hLH. For all 113 patients, cumulus cells from all oocytes were denuded/collected individually on the day of oocyte retrieval followed by ICSI.

### Patient characteristics and embryology and clinical outcomes

This study aimed to identify potential biomarkers predictive for oocyte competence in two patient groups undergoing ART. To assess the applicability of a common set of biomarkers across both groups, the expression of five key biomarkers was initially compared. To ensure comparability, patient characteristics and stimulation protocols were compared (Table 1).

**Table 1:**
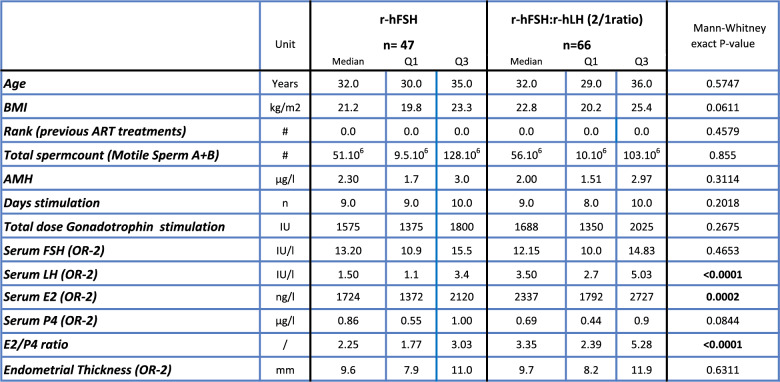
Patient characteristics & stimulation in the two patient groups

Both patient groups were compared with Mann-Whitney tests

Bonferroni corrected p values < 0.0025 were significant

The ages of the patients were 32 in both the r-hFSH group and in the r-hFSH: r-hLH group (Table 1). All participants underwent their first or second ART cycle. For at least 75% of the patients it was their first cycle (Q3 = 0 in Table 1) in both patient groups. SET was scheduled on day 5 post-ICSI for all patients.

In both treatment groups, similar r-hFSH doses were administered, resulting in comparable serum FSH levels (Table 1). However, in the r-hFSH: r-hLH patient group, significantly higher serum LH levels (*p* < 0.0001) and oestradiol (E₂) concentrations (*p* = 0.0002) were observed two days prior to oocyte retrieval. Additionally, the E2/P4 ratio differed significantly between the two groups (*p* < 0.0001) (Mann–Whitney test, Table 1).

Embryology outcomes are summarized in Table 2. Clinical outcomes are detailed in Table 3. No significant differences were observed between the two patient groups regarding the βhCG rate, clinical pregnancy rate, ongoing clinical pregnancy rate, and live birth rate. These comparative analyses of clinical parameters, embryology and clinical outcomes were conducted as post hoc analyses, as detailed in Tables 1, 2 and 3.

**Table 2:**
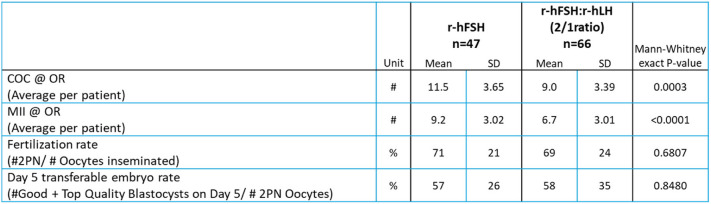
Embryology results in the two patient groups

Mean maturation (MII), fertilization and day 5 transferable embryo rates were calculated per patient

**Table 3:**
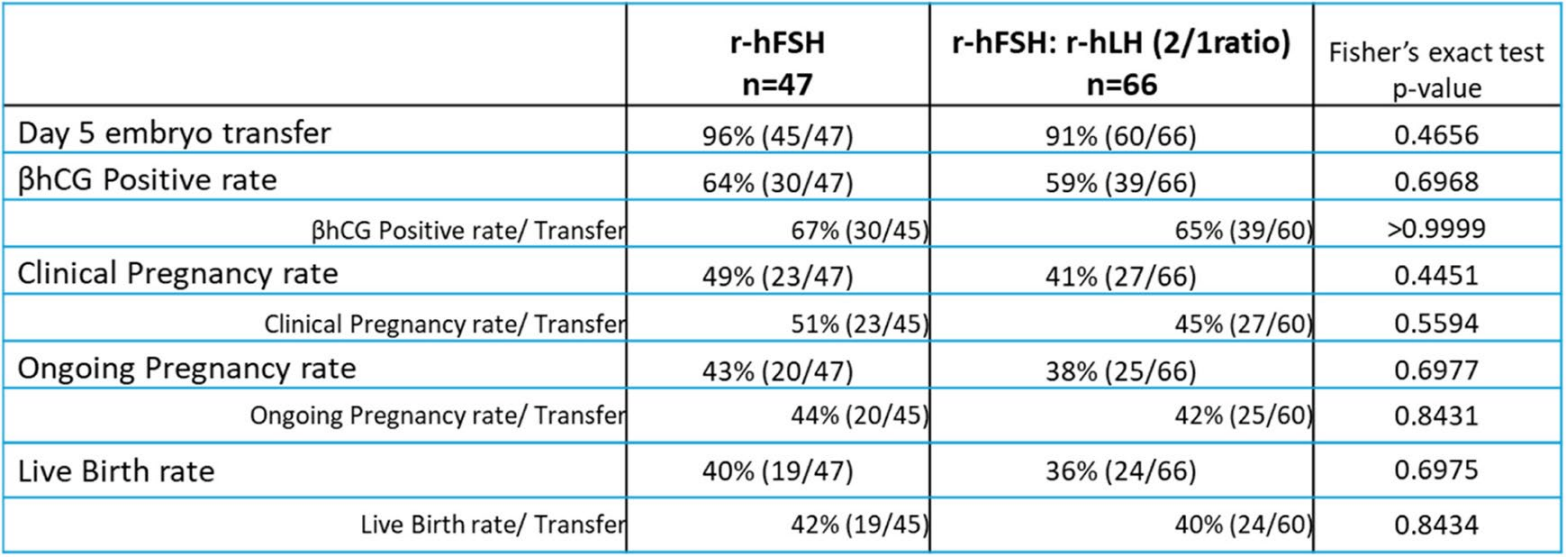
Outcome of the fresh single embryo transfer in the two patient groups

Differences between the two patient groups were not significant

### Correlation analysis of patient characteristics

To minimize potential bias between the two patient groups, correlations between clinical patient characteristics and their gene expression data were analysed. In the r-hFSH stimulated group, Spearman correlation analysis revealed a significant positive correlation between serum LH and serum E2 (Spearman *r* = 0.54 and *p* < 0.0001) (Supplementary Table 1a). Conversely, in the r-hFSH: r-hLH group, a significant negative correlation was observed between serum AMH and total FSH dose (*r* = −0.55; *p* < 0.0001 (Supplementary Table 1b). These differing correlations may suggest variations in the two ovarian stimulation protocols r-hFSH and r-hFSH: r-hLH and the resulting endocrine environment between the two groups.

### Gene expression comparison between r-hFSH and r-hFSH: r-hLH treatment protocols using 5 biomarker genes

**Fig. 2:**
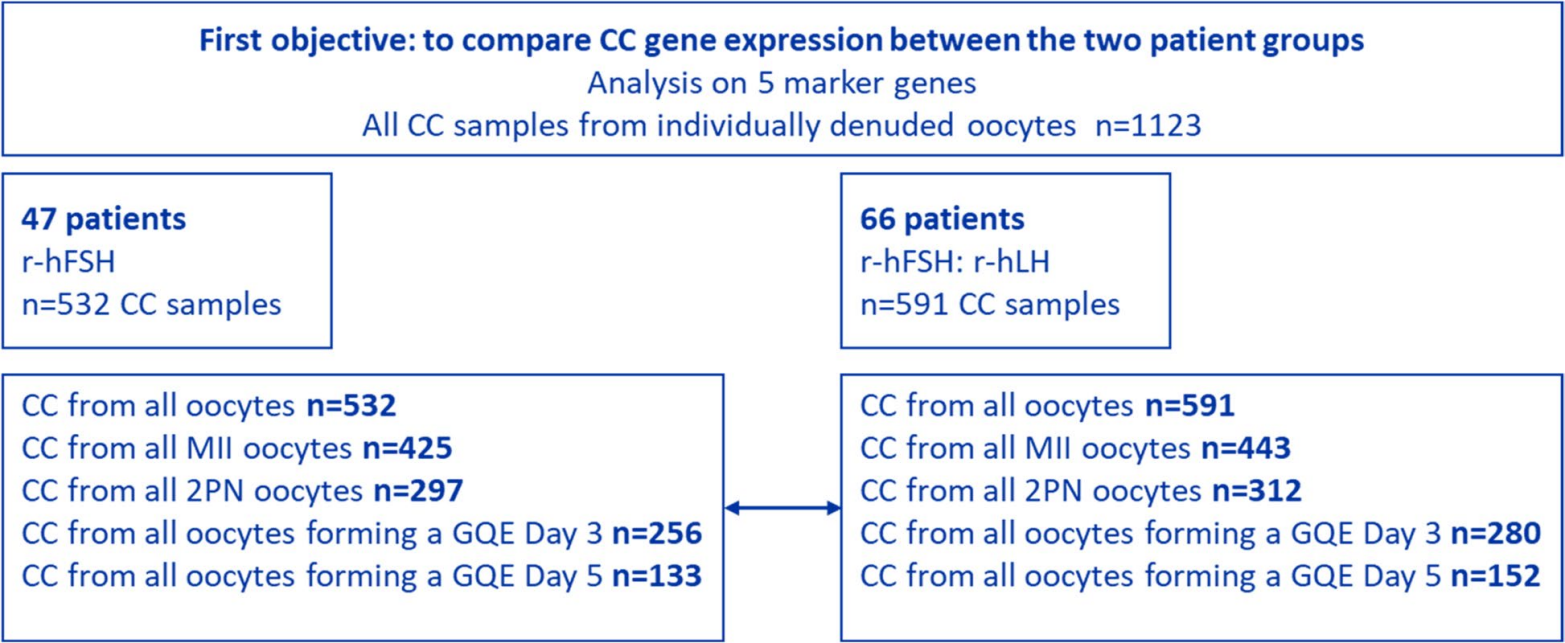
Sample inclusion and analysis flowchart for both patient groups for the first study objective to compare CC gene expression between the two patient groups. Each subgroup was compared with the other, in total 5 comparisons

In the initial phase of the gene expression analysis, 12 out of 1135 CC samples (1%) were excluded due to too low gene expression in at least two of the studied genes. Consequently, 1123 CC samples (532 from the r-hFSH group and 591 from the r-hFSH: r-hLH group) were retained for further analysis, as depicted in the sample inclusion flowchart (Fig. 2).

The expression of five key biomarker—EFNB2, CAMK1D, SASH1, GOT1 and SLC6A9—was assessed across five datasets: (i) CC of all oocytes, (ii) CC of all MII oocytes, (iii) CC of all 2PN oocytes, (iv) CC of all oocytes forming a GQE day 3, and (v) CC of all oocytes forming a GQE day 5 (Fig. 2).

**Fig. 3:**
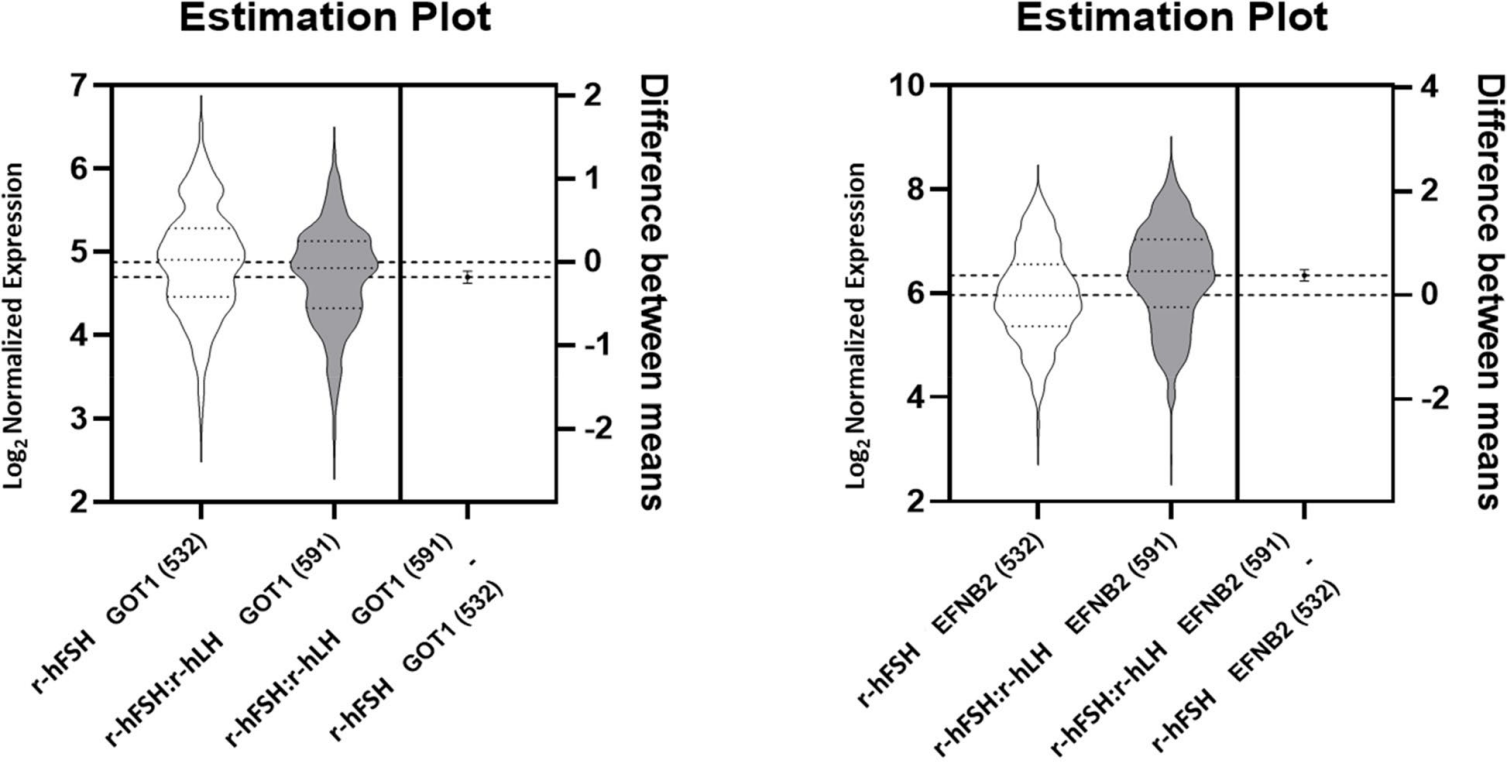
Estimation plot depicting the expression difference observed when comparing the CC of all oocytes available in both stimulation groups. Violins represent all samples, thin dotted lines represent 25%, 50% (median), 75% quartiles and thick dotted lines represent means. Error bars depict 95%CI

Detailed one-parametric analysis results of these five biomarker genes in the five sample sets are shown in Table 4. No statistically significant differences were observed in gene expression for CAMK1D, SASH1 and SLC6A9 between the two stimulation protocols across all sample groups. However, EFNB2 exhibited significantly higher expression in the r-hFSH: r-hLH group compared to the r-hFSH group, while GOT1 showed lower expression in the r-hFSH: r-hLH group (Table 4, Fig. 3).

**Table 4:**
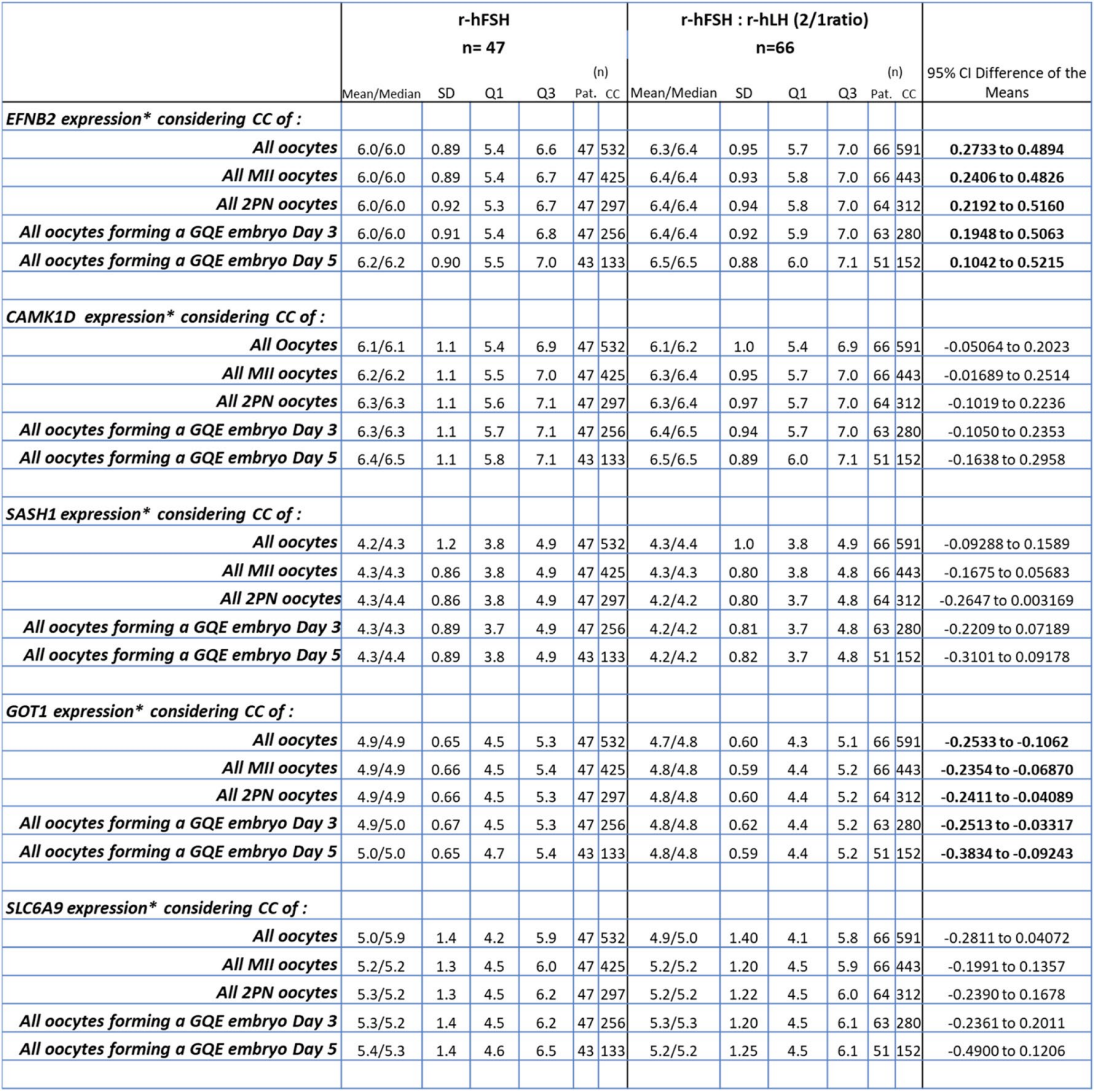
One-parametric analysis of the gene expression of five biomarkers in CC of r-hFSH and r-hFSH: r-hLH stimulated patients

Significant results are shown in bold

*All expression values were normalized for the expression of the endogenous controls and log2 transformed

To further investigate the differential expression of the key five biomarkers—EFNB2, CAMK1D, SASH1, GOT1, and SLC6A9 – between the two treatment groups, a multiparametric analysis using MANOVA in R. This approach was applied to two a multiparametric analysis was performed datasets: (i) CC of all oocytes (largest sample set) and (ii) CC of all oocytes forming a GQE on day 5 (most selective sample set). The results of this analysis corroborated the findings from the univariate (one-parametric) analyses, confirming the differential expression patterns observed for EFNB2 and GOT1 between the two treatment groups. For detailed statistical outcomes and further insights, please refer to Supplemental Tables 2a and b.

### Gene expression correlations between clinical variables and 11 biomarker genes

In both the r-hFSH and the r-hFSH: r-hLH treatment groups, no strong correlations were observed between the clinical variables and the expression of the 11 biomarker genes analysed (Supplementary Table 3a and b). This suggests that clinical factors did not significantly influence gene expression levels, ensuring that observed gene expression patters are not confounded by clinical variables.

### Gene expression correlation among biomarker genes

Within each treatment group, several significant correlations were identified among the 11 biomarker genes. In the r-hFSH group, eight significant correlations were found between: SASH1 and CAMK1D (*r* = 0.54; *p* < 0.0001), EFNB2 and HAS2 (*r* = 0.68; *p* < 0.0001), EFNB2 and VCAN (*r* = 0.60; *p* < 0.0001), GOT1 and SLC6A9 (*r* = 0.51; *p* = 0.0002), GOT1 and STC2 (*r* = 0.69; *p* < 0.0001), GOT1 and GSTA4 (*r* = 0.60; *p* < 0.0001), SLC6A9 and STC2 (*r* = 0.50; *p* = 0.0004) and HAS2 and VCAN (*r* = 0.56; *p* < 0.0001).

In the r-hFSH: r-hLH group, ten significant correlations were found between: CAMK1D and EFNB2 (*r* = 0.55; *p* < 0.0001), CAMK1D and PTGS2 (*r* = 0.50; *p* < 0.0001), EFNB2 and HAS2 (*r* = 0.74; *p* < 0.0001), EFNB2 and PTGS2 (*r* = 0.51; *p* < 0.0001), EFNB2 and VCAN (*r* = 0.71; *p* < 0.0001), GOT1 and STC2 (*r* = 0.54; *p* < 0.0001), GOT1 and HSPH1 (*r* = 0.59; *p* < 0.0001), GOT1 and GSTA4 (*r* = 0.66; *p* < 0.0001), HAS2 and VCAN (*r* = 0.60; *p* < 0.0001) and PTGS2 and VCAN (*r* = 0.59; *p* < 0.0001).

### Constructing models using 11 biomarker genes predicting live birth

For the secondary study objective, gene expression was analysed on CC from transferred blastocysts in both patient groups to identify the most effective combination of biomarkers, predictive for live birth. In total, 146 CC samples associated with transferred (fresh and frozen transfers) blastocysts were analysed for expression levels of 11 biomarker genes.

Multiparametric model building was performed using gene expression data from the following 11 genes: CAMK1D, EFNB2, SASH1, GOT1, SLC6A9, HAS2, PTGS2, HSPH1, VCAN, GSTA4, STC2. Interpatient comparisons were conducted with the endpoint of live birth. The analysis aimed to distinguish gene expression patterns in CCs associated with transferred blastocysts that did or did not result in a live birth.

**Fig. 4:**
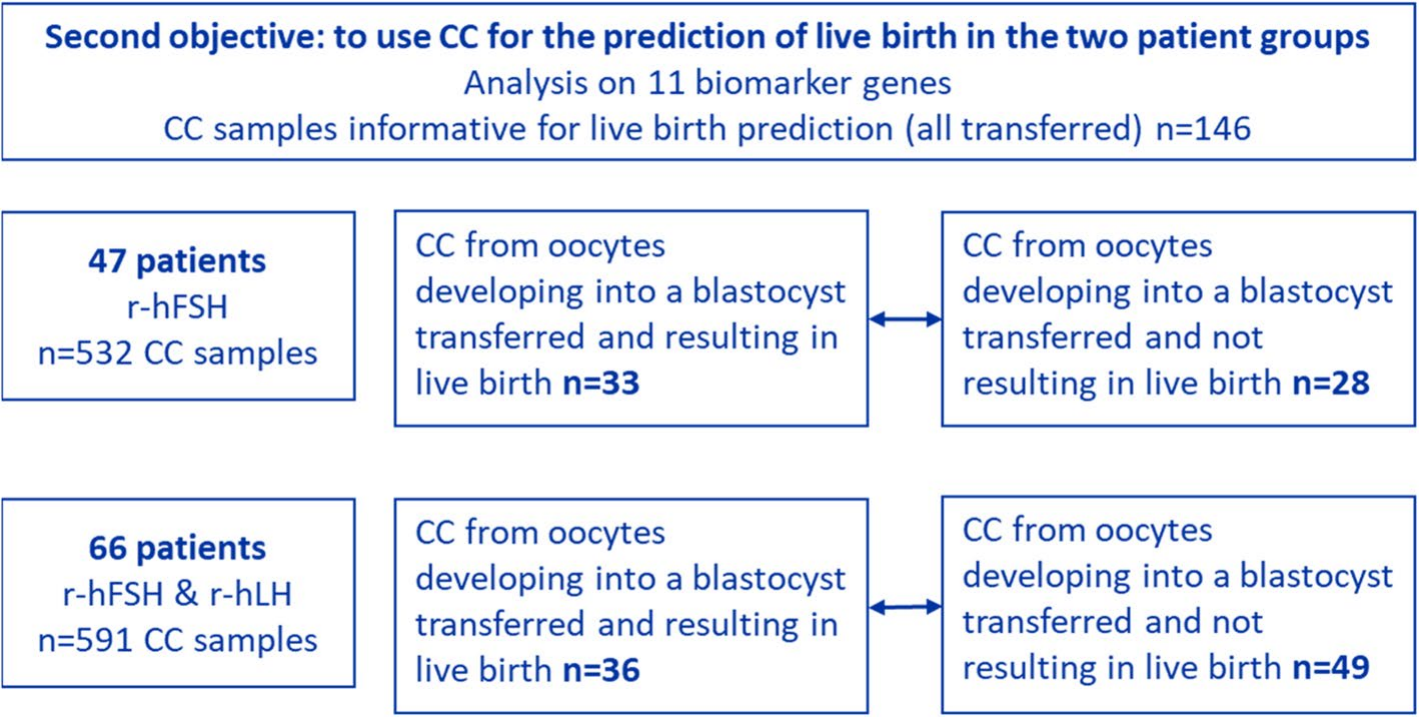
Sample inclusion and analysis flowchart for both patient groups for the second study objective to use CC for the prediction of live birth in the two patient groups

In the group of patients stimulated with r-hFSH, gene expression of 33 CC samples linked to blastocysts that resulted in a live birth was compared with 28 CC samples linked to blastocysts that did not. In the group stimulated with r-hFSH: r-hLH, 36 CC samples associated with live birth were compared with 49 with non-live birth outcomes (Fig. 4).

To identify the most potent biomarkers, a “leave-one-out cross-validation” approach was applied—61 iterations in the r-hFSH group, and 85 iterations in the r-hFSH: r-hLH patient group. Table 5 presents the frequency with which each biomarker gene was selected as one of the top predictors across these iterations. Biomarkers ranked higher in frequency were considered more relevant within the respective patient group. In the r-hFSH group, GOT1, HAS2, SASH1 and PTGS2 emerged as the most predictive genes for live birth. In the r-hFSH: r-hLH group, GOT1, HAS2, EFNB2, STC2 and GSTA4 were identified as key predictors. Notably, GOT1 ranked at the top predictor in both stimulation protocols, followed closely by HAS2 (Table 5).

**Table 5:** “Leave-one-out” cross-validation for the r-hFSH patient group (*n* = 61 CC from transferred oocytes) and the r-hFSH: r-hLH patient group (*n* = 85 CC from transferred oocytes) to identify the strongest biomarkers

r-hFSH patient group (*n* = 61)		r-hFSH: r-hLH patient group (*n* = 85)	
**Gene**	**Frequency of occurrence**	**Gene**	**Frequency of occurrence**
GOT1	38	GOT1	47
HAS2	38	HAS2	43
SASH1	38	EFNB2	40
PTGS2	37	STC2	26
HSPH1	1	GSTA4	23
		VCAN	7
		SASH1	2

Following this, stepwise linear regression was employed to develop predictive models evaluating multiple combinations of up to four biomarkers in interpatient comparisons. The best performing models predictive of live birth are summarized in Table 6 (for r-hFSH group) and Table 7 (for r-hFSH: r-hLH group).

**Table 6:**
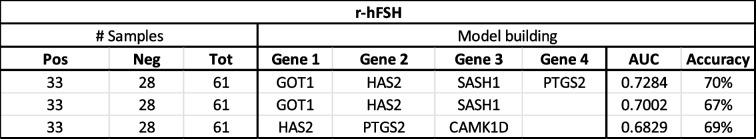
Overview of the stepwise linear regression model building for the r-hFSH patient group showing the number of samples, the genes and the predictive value of the live birth models with ROC analysis, showing the area under the curve (AUC) and accuracy of the obtained live birth predictive models

**Table 7:**
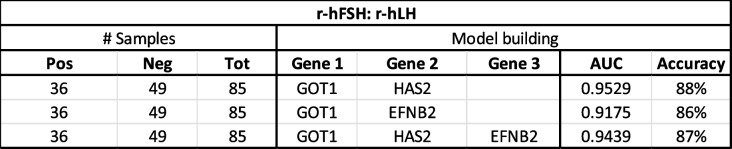
Overview of the stepwise linear regression model building for the r-hFSH: r-hLH patient group showing the number of samples, the genes and the predictive value of the live birth models with ROC analysis, showing the area under the curve (AUC) and accuracy of the obtained live birth predictive models

**Fig. 5:**
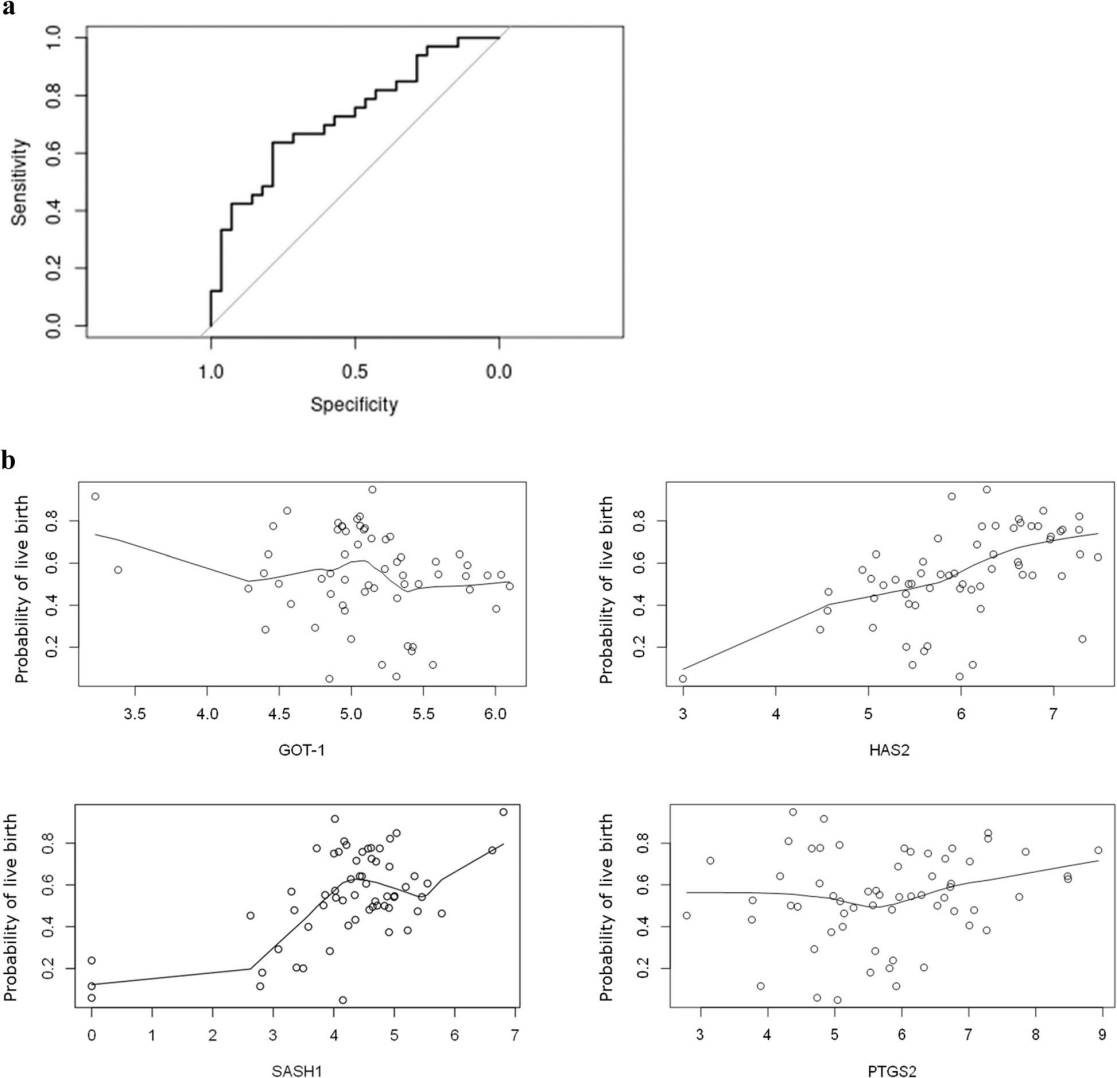
**a** ROC (Receiver-operating characteristic) curve for the live birth predictive model with GOT1, HAS2, SASH1 and PTGS2 in the r-hFSH patient group with AUC (area under the curve) of 0.7284 and an accuracy of 70%. **b** Relations between the gene expression of GOT1, HAS2, SASH1 and PTGS2 and the predicted outcome in r-hFSH patients. These graphs are a two-dimensional representation of the relation of one gene with the probability of live birth, while abstracting the other influencing parameters

In the r-hFSH group, the optimal model included GOT1, HAS2, SASH1 and PTGS2 achieving an AUC of 0.7284 and an accuracy of 70% (Fig. 5a and b).

**Fig. 6:**
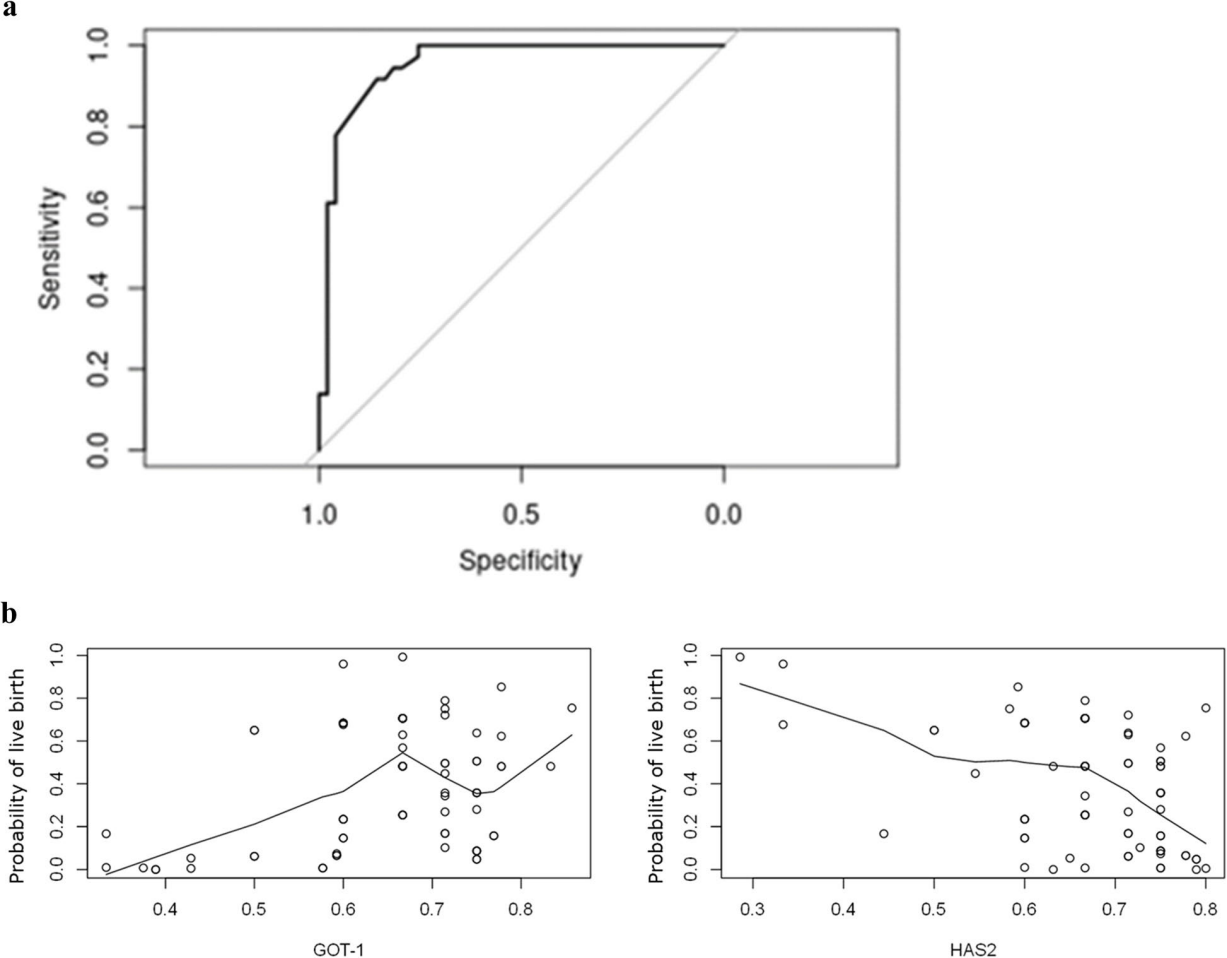
**a** ROC (Receiver-operating characteristic) curve for a live birth predictive model with GOT1 and HAS2 in r-hFSH: r-hLH patients with an AUC (area under the curve) of 0.9529 and an accuracy of 88%. **b** Relations between the gene expression of GOT1 and HAS2 and the predicted outcome in r-hFSH: r-hLH patients. These graphs are a two-dimensional representation of the relation of one gene with the probability of live birth, while abstracting the other influencing parameters

In the r-hFSH: r-hLH group, the most predictive model was a two-gene model including GOT1 and HAS2, with an AUC of 0.9529 and an accuracy of 88% (Fig. 6a and b).

To assess the potential clinical impact of the newly developed CC-based predictive gene expression models in a standard-of-care context, the models were retrospectively applied to patients in the current study who had multiple good or top-quality embryos available for transfer. In this retrospective application, the CC-based models would have suggested a different top/good-quality blastocyst for the first transfer in 32% of r-hFSH patients (12 out of 37) and 63% of r-hFSH: r-hLH patients (27 out of 43). 

## Discussion

In this study, cumulus cell (CC) gene expression was systematically analysed for the first time in patients undergoing ICSI and day 5 embryo transfer following two types of ovarian stimulation protocols: one using recombinant follicle stimulating hormone alone (r-hFSH, Gonal-*f*: follitropin alfa), and the other combining recombinant follicle stimulation hormone with recombinant luteinizing hormone (r-hFSH: r-hLH, Pergoveris: combination of follitropin alfa and lutropin alfa).

Among five preselected biomarker genes, two (GOT1 and EFNB2) were found to be significantly differently expressed between these two ovarian stimulation protocols. GOT1 expression was consistently higher in CC from r-hFSH-stimulated patients compared to those of the r-hFSH: r-hLH group. This pattern was observed when considering CC of oocytes with different developmental capacities, including all retrieved oocytes, mature MII oocytes, fertilized 2PN oocytes and embryos that developed to day 3 and day 5. In contrast, EFNB2 expression was lower in the r-hFSH group compared to the r-FSH: r-hLH group across the same sets of CC samples.

These findings suggest that the presence of recombinant LH in the stimulation regimen has a measurable impact on the transcriptomic profile of cumulus cells. Given that two out of five studied genes show significant differential expression, it is likely that genome-wide transcriptomic analysis would identify a much broader set of differentially expressed genes between the two stimulation protocols.

The observed differences are presumably driven by the biological actions of human luteinizing hormone (hLH), which is included in the second stimulation protocol but absent in the r-hFSH-alone regime. hLH plays essential roles in follicular recruitment and maturation, the resumption of meiosis, extrusion of the first polar body and decidualization of endometrial stromal cells, all of which are critical for embryo implantation [34]. Why the role of r-hLH in ovarian stimulation (OS) across a diverse infertility profile remains a topic of ongoing debate, there is growing evidence supporting its utility in specific patient populations [35, 36].

Although the clinical characteristics and stimulation regimens were similar between the two ovarian stimulation groups, significant differences were observed in their hormonal profiles. In the r-hFSH:r-hLH group, serum levels of LH (*p* < 0.0001) and oestradiol (E2; *p* = 0.0002) were significantly higher two days prior to oocyte retrieval compared to the r-hFSH-only group. The E2/P4 ratio was also significantly different (*p* < 0.0001). These findings are consistent with earlier studies comparing r-hFSH and HP-hMG stimulation protocols [37]. Whether the additional r-hLH in the r-hFSH:r-hLH group has the same clinical and molecular effects as the urinary hCG present in HP-hMG (e.g., Menopur) remains to be determined.

Furthermore, in the r-hFSH:r-hLH group, a significant negative correlation was found between serum AMH levels and the total FSH dose administered (*r* = −0.55; *p* < 0.0001), which is expected as lower AMH levels reflect reduced follicular reserve and increased gonadotropin requirements. No significant correlation was observed between serum LH and E2 concentrations (*r* = 0.23; *p* = 0.06), likely due to the presence of exogenous LH in the stimulation regimen.

The fact that human luteinizing hormone is changing the expression signature in cumulus cells of patients receiving OS with r-hFSH is in analogy with previous results that gene expression in cumulus differs in women stimulated with highly purified urinated human menopausal gonadotropin (HP-hMG, Menopur) or with r-hFSH [17–19]. Here, one major difference between HP-hMG and r-hFSH is the presence of human chorionic gonadotropin in the first.

The differential expression of GOT1 and EFNB2 in response to stimulation protocols differing only in the presence of r-hLH is particular compelling, as both genes have already been linked to oocyte competence in independent studies [15, 23, 32, 33] as well as our own unpublished micro array and qPCR data. Notably, both genes were also retained as predictive biomarkers in our live birth prediction models.

EFNB2, a transmembrane ligand in the ephrin family, is predominantly expressed during luteinization in granulosa cells [38]. Its receptor, EPHB2, is regulated by FSH and the oestrogen receptor beta [39]. Increased EFNB2 expression may promote improved follicular vascularization [40] thereby supporting oocyte competence. Moreover, expression of the EFNB2 receptor in the CCs has also been associated with oocyte/embryo competence and euploidy [41].

GOT1 (glutamic-oxaloacetic transaminase 1) plays a central role in the malate-aspartate shuttle, converting glutamate to aspartate using oxaloacetate. This metabolic process supports redox homeostasis and NAD + regeneration during oocyte maturation [42]. Aspartate, a non-essential amino acid, is among the most consumed amino acid in mouse, cow, and pig blastocysts [43–47]. Increased GOT1 expression may therefore enhance cellular redox status and NAD + availability, contributing to oocyte development potential [45, 48, 49].

While the exact mechanism underlying the differential CC expression of GOT1 and EFNB2 in response r-hLH exposure is currently unclear, our findings add to the growing body of evidence supporting the functional importance of gonadotropin-induced transcriptional changes in CCs and their potential utility in improving embryo selection strategies.

As a next step, predictive gene expression models for live birth were systematically constructed using a panel of 11 biomarker genes and evaluated for their performance using linear regression methods. Model performance was assessed based on area under the curve (AUC) and overall accuracy. Given the previously observed difference in gene expression profiles between the two ovarian stimulation protocols, a stimulation group-specific approach was adopted for model development. The 11 genes evaluated were CAMK1D, EFNB2, SASH1, GOT1, SLC6A9, HAS2, PTGS2, HSPH1, VCAN, GSTA4, and STC2, all previously identified as expression biomarkers informative of oocyte competence. For model construction, gene expression data from cumulus cells of oocytes that developed into blastocysts and resulted live births were compared to those from blastocysts that failed to yield live births after transfer.

‘Leave-one-out’ cross validation identified the most frequently occurring genes in predictive models. In the r-hFSH-alone group, the most consistently selected genes were GOT1, HAS2, SASH1, and PTGS2 (Table 5). In the r-hFSH: r-hLH group, the top genes were GOT1, HAS2, EFNB2, STC2, GSTA4 and VCAN (Table 5).

The best performing model for r-hFSH patient group consisted of four genes (GOT1, HAS2, SASH1, PTGS2), yielding an AUC of 0.7284 and an overall accuracy of 70%. In the r-hFSH: r-hLH group, the optimal model was a two-gene combination (GOT1 and HAS2), achieving an AUC of 0.9529 and an overall accuracy of 88%. The superior performance in the r-hFSH: r-hLH group likely reflects the availability of approximately 30% more informative samples (CCs of transferred embryos with known outcomes) in that group.

Correlation analysis of gene expression data further informed model refinement by identifying genes that likely function within shared regulatory pathway. Genes showing high co-correlation are less suitable for inclusion together in a predictive model due to potential redundancy. For instance, while GOT1 correlated with three other genes, it was not correlated with HAS2 and EFNB2, supporting their combined inclusion in predictive models. Additionally, three gene pairings (GOT1 vs HPSH1; SASH1 vs CAMK1D; SLC6A9 vs STC2) exhibited differing correlation patterns between the two patient groups, potentially reflecting stimulation protocol-specific effects (e.g. elevated GOT1 expression in the r-hFSH group).

GOT1 and HAS2 were among the strongest predictors of live birth across both stimulation protocols. However, the correlation of HAS2 with live birth varied by group, indicating protocol-dependent dynamics. A similar phenomenon has been described previously for PTGS2, where studies reporting opposing associations with oocyte competence depending on whether patients were stimulated with rFSH [50] or HP-hMG which includes both FSH and LH components [10]. This apparent contradiction was further validated in a comparative study involving both patient populations [17], underscoring the need for stimulation-specific predictive models in CC-based analysis.

HAS2 encodes hyaluronan synthase 2, an enzyme critical for hyaluronan acid production and cumulus cell expansion, processes that are crucial during oocyte maturation. HAS2 is upregulated in response to GDF-9, a key oocyte-secreted factor, and supports the structural remodelling required for optimal oocyte function [51, 52]. Previous work by our group linked HAS2 expression in CCs to increased rates of 2PN fertilization, suggesting it may serve as a marker of oocyte maturity or over maturity [17].

SASH1 (SAM and SH3 domain-containing protein 1) belongs to the SLY1-family of signal adapter proteins. Unlike most family members which are haematopoietic, SASH1 is ubiquitously expressed including in the ovary, uterus, placenta, prostate and testis. This gene is also included in the Aurora Test, a pregnancy prediction tool prospectively validated for HP-hMG-stimulated patients [15, 16]. SASH1 has been implicated in the Toll-like receptor 4 pathway [53] and is known to be LH-responsive in bovine granulosa cells [54].

PTGS2 (prostaglandin-endoperoxide synthase 2) has long been associated with oocyte maturation and cumulus expansion and has been identified as predictive CC biomarker by multiple research groups [10, 13, 55–57]. Its inclusion in the predictive model for the r-hFSH group reinforces its value in the context of FSH-driven folliculogenesis.

Taken together, these stimulation-specific predictive biomarkers reflect the diverse molecular pathways involved in cumulus-oocyte signalling and highlight distinct mechanisms through which a competent oocyte and, ultimately, a viable embryo can be generated to result in clinical pregnancy and live birth.

The CC based live birth prediction models developed here would have suggested a different top/good-quality blastocyst for the first transfer in 32% of r-hFSH patients of the current study. This finding aligns with previous observations from an unbiased, interventional prospective study using the Aurora Test in HP-hMG stimulated patients, where only 27% concordance was found between embryo ranking based on morphology and ranking based on CC gene expression analysis. In that study prioritizing the transfer of day 3 embryos based on the CC analysis resulted in a doubling of the pregnancy rate compared to the controls with transfer based on morphology only [15, 16]. This confirms the impact a CC based oocyte/embryo grading can have on routine ART.

### Study limitations

This clinical study was an observational cohort study and not a randomized clinical trial and was conducted between 2021 and 2023 at a single tertiary university IVF centre. Patients were enrolled until at least 20 patients with a confirmed ongoing pregnancy were included in both study groups, which was necessary to support the development of predictive models for the secondary study objective. One limitation is that equal recruitment across study arms was not achieved. Ultimately, 19 more patients were included in the r-hFSH: r-hLH arm. It remains unclear whether the observed difference between the two patient groups in the number of COC and MII oocytes between the two groups reflect a clinically relevant effect, a difference in the baseline characteristics or simply a random variation due to the limited patient number.

The CC gene expression-based prediction models developed here are intended to be used in conjunction with standard morphological blastocyst grading. Efforts to develop models that predict oocyte competence independently of morphologic grading are currently ongoing.

This study should be regarded as a discovery study. While the gene expression models demonstrated high predictive accuracy, particularly in both ovarian stimulation protocols, the findings are based on a non-randomized, single-centre design and should be interpreted accordingly.

Implementation of CC-based gene expression analysis in clinical practice would require individual denudation of oocytes, which is not current standard practice. While oocyte denudation is typically performed in groups for efficiency, individual denudation adds approximately 30 min per patient, representing a manageable but relevant increase in embryology lab workload.

Finally, while several of the biomarker genes used in the new predictive models such as GOT1, HAS2, SASH1 and PGTS2 have been previously associated with oocyte competence or cumulus function, their exact mechanistic roles remain to be fully elucidated. Although a complete understanding of their function is not necessary for the utilization of the predictive models, further functional studies would strengthen the biological foundation.

## Conclusions

This study identified novel cumulus cell gene expression models that predict oocyte competence and are associated with live birth outcomes in patients undergoing ART. Biomarker expression profiles differed between patients stimulated with r-hFSH alone and those receiving r-hFSH in combination with r-hLH, underscoring the need for stimulation-specific predictive approaches. The most robust gene models achieved an AUC between 0.72 and 0.95, with an overall accuracy for predicting live birth ranging from 70 to 88%. Given their potential to improve embryo selection, increase live birth per transfer and shorten the time-to-pregnancy in ICSI cycles, these models merit validation in prospective, randomized clinical trials.

## Supplementary Information

Supplementary Material 1. Supplementary Table 1a: Spearman correlation analysis for patient characteristics in the r-hFSH stimulated group. Correlations with Spearman *r* >0.5 or <-0.5 and *p* < 0.0023 were highlighted. Supplementary Table 1b: Spearman correlation analysis for patient characteristics in the r-hFSH: r-hLH stimulated group. Correlations with Spearman *r* >0.5 or <-0.5 and *p* < 0.0023 were highlighted. Supplementary Table 2a and b: The analysis was repeated in a multiparametric approach (MANOVA, using R) for the largest (*n*=1123, Table 2a) and the smallest sample set (*n*=285, Table 2b). The results obtained for “CC of all oocytes (*n*=1123)” and “CC of all oocytes forming a GQE on day 5 (*n*=285)” were comparable. The expression in the two treatment groups was statistically different. This difference was detectable for EFNB2 and GOT1 but also for CAMK1D and SLC6A9. The largest differences observed were between 0.5 and 0.75. As this were log2 values actual differences were between 1.4-1.7 fold or 40% to 70% higher/lower expression between the two stimulation groups. Supplementary Table 2a: MANOVA results when comparing the CC gene expression in “All oocytes” sample set. *P* values are enumerated in the columns for 6 hypothesis (difference between the 2 groups ><0, <0.1, <0.5, <0.75, <1, <1.5log2), and the analysis was repeated for all combinations comprising 1, 2, 3, 4 or 5 gene expressions. All analyses were performed in R. The green line (indicating significance) is added for the ease of interpretation of the table. The first column detects difference between the 2 treatment groups. If this is 0=significant then the 5 columns to the right are considered. These allow an estimation on how large the difference is between the 2 treatments. Blue arrows indicate the largest differences observed when considering the expression of only 1 or 2 or 3 genes. Supplementary Table 2b: MANOVA results when comparing the CC gene expression in “All oocytes forming a GQE on day 5” sample set. *P* values are enumerated in the columns for 6 hypothesis (difference between the 2 groups ><0, <0.1, <0.5,<0.75, <1, <1.5 log2), and the analysis was repeated for all combinations comprising 1, 2, 3, 4 or 5 gene expressions. All analyses were performed in R. The green line (indicating significance) is added for the ease of interpretation of the table. The first column detects difference between the 2 treatment groups. If this is 0=significant then the 5 columns to the right are considered. These allow an estimation on how large the difference is between the 2 treatments. Blue arrows indicate the largest differences observed when considering the expression of only 1 or 2 or 3 genes. Supplementary Table 3a: Spearman correlation analysis for patient characteristics and gene expression data in the r-hFSH stimulated group. Correlations with Spearman *r* >0.5 or <-0.5 and *p* < 0.0023 were highlighted and discussed in detail. Supplementary Table 3b: Spearman correlation analysis for patient characteristics and gene expression data in the r-hFSH: r-hLH stimulated group. Correlations with Spearman *r* >0.5 or <-0.5 and *p* < 0.0023 were highlighted and discussed in detail.

## Data Availability

All data are incorporated into this article and its online supplementary material.

## Abbreviations

2PN: Two pronuclei
AFS: American Fertility Society
AI: Artificial intelligence
AMH: Anti-Mullerian hormone
ART: Assisted reproductive technologies
ASRM: American Society for Reproductive Medicine
AUC: Area under the curve
B2M: Beta-2-Microglobulin
BMI: Body mass index
CAMK1D: Calcium/Calmodulin Dependent Protein Kinase ID
CC: Cumulus cells
cDNA: Complementary DNA
CI: Confidence interval
COC: Cumulus oocyte complex
DET: Double embryo transfer
E2: Oestradiol
EFNB2: Ephrin B2
ESHRE: European Society of Human Reproduction and Embryology
GLMM: Generalized linear mixed model
GnRH: Gonadotrophin releasing hormone
GOT1: Glutamic-Oxaloacetic Transaminase 1
GQE: Good quality embryo
GSTA4: Glutathione S-Transferase Alpha 4
HAS2: Hyaluronan Synthase 2
hCG: Human choriononic gonadotrophin
hLH: Human luteinizing hormone
HSPH1: Heat Shock Protein Family H (Hsp110) Member 1
ICSI: Intracytoplasmic sperm injection
IVF: In-vitro fertilization
LOWESS: Locally Weighted Scatterplot Smoothed
MANOVA: Multivariate analysis of variance
MII: Metaphase II
OAT: Oligo-astheno-teratozoospermia
OPR: Ongoing pregnancy rate
OR: Oocyte retrieval
PCOS: Polycystic ovary syndrome
PGT-A: Preimplantation Genetic Testing for aneuploidy
PTGS2: Prostaglandin-Endoperoxide Synthase 2
Q-Q: Quantile–quantile
RCT: Randomized controlled trial
r-hFSH: Recombinant human follicle stimulating hormone
r-hLH: Recombinant human luteinizing hormone
RNA: Ribonucleic acid
RT-qPCR: Reverse Transcription quantitative Polymerase Chain Reaction
SASH1: SAM and SH3 domain-containing protein 1
SET: Single embryo transfer
SLC6A9: Solute Carrier Family 6 Member 9
STC2: Stanniocalcin 2
TESE: Testicular sperm extraction
UBC: Ubiquitin C
VCAN: Versican

## References

[1] Practice Committees of the American Society for Reproductive Medicine and the Society for Assisted Reproductive Technology. Electronic address: asrm@asrm.org. The use of preimplantation genetic testing for aneuploidy: a committee opinion. Fertil Steril. 2024;122(3):421–34. 38945424 10.1016/j.fertnstert.2024.06.021

[2] Munné S, Kaplan B, Frattarelli JL, Child T, Nakhuda G, Shamma FN, et al. Preimplantation genetic testing for aneuploidy versus morphology as selection criteria for single frozen-thawed embryo transfer in good-prognosis patients: a multicenter randomized clinical trial. Fertil Steril. 2019;112(6):1071-1079.e7. 31551155 10.1016/j.fertnstert.2019.07.1346

[3] Simopoulou M, Sfakianoudis K, Maziotis E, Tsioulou P, Grigoriadis S, Rapani A, et al. PGT-A: who and when? A systematic review and network meta-analysis of RCTs. J Assist Reprod Genet. 2021;38(8):1939–57. 34036455 10.1007/s10815-021-02227-9 PMC8417193

[4] Pirtea P, De Ziegler D, Tao X, Sun L, Zhan Y, Ayoubi JM, et al. Rate of true recurrent implantation failure is low: results of three successive frozen euploid single embryo transfers. Fertil Steril. 2021;115(1):45–53. 33077239 10.1016/j.fertnstert.2020.07.002

[5] Sakkas D, Navarro-Sánchez L, Ardestani G, Barroso G, Bisioli C, Boynukalin K, et al. The impact of implementing a non-invasive preimplantation genetic testing for aneuploidies (niPGT-A) embryo culture protocol on embryo viability and clinical outcomes. Hum Reprod. 2024;39(9):1952–9. 39059790 10.1093/humrep/deae156

[6] Navarro-Sánchez L, García-Pascual C, Rubio C, Simón C. Non-invasive preimplantation genetic testing for aneuploidies: an update. Reprod Biomed Online. 2022;44(5):817–28. 35307298 10.1016/j.rbmo.2022.01.012

[7] Jiang VS, Bormann CL. Artificial intelligence in the *in vitro* fertilization laboratory: a review of advancements over the last decade. Fertil Steril. 2023;120(1):17–23. 37211062 10.1016/j.fertnstert.2023.05.149

[8] Armstrong S, Bhide P, Jordan V, Pacey A, Marjoribanks J, Farquhar C. Time-lapse systems for embryo incubation and assessment in assisted reproduction. Cochrane Database Syst Rev. 2019 May 29;5(5):CD011320. 10.1002/14651858.CD011320.pub4 PMC6539473 31140578

[9] Zou H, Wang R, Morbeck DE. Diagnostic or prognostic? Decoding the role of embryo selection on in vitro fertilization treatment outcomes. Fertil Steril. 2024;121(5):730–6. 38185198 10.1016/j.fertnstert.2024.01.005

[10] McKenzie LJ, Pangas SA, Carson SA, Kovanci E, Cisneros P, Buster JE, et al. Human cumulus granulosa cell gene expression: a predictor of fertilization and embryo selection in women undergoing IVF. Hum Reprod. 2004;19(12):2869–74. 15471935 10.1093/humrep/deh535

[11] Patrizio P, Fragouli E, Bianchi V, Borini A, Wells D. Molecular methods for selection of the ideal oocyte. Reprod Biomed Online. 2007;15(3):346–53. 17854537 10.1016/s1472-6483(10)60349-5

[12] Conti M, Franciosi F. Acquisition of oocyte competence to develop as an embryo: integrated nuclear and cytoplasmic events. Hum Reprod Update. 2018;24(3):245–66. 29432538 10.1093/humupd/dmx040 PMC5907346

[13] Wathlet S, Adriaenssens T, Segers I, Verheyen G, Van de Velde H, Coucke W, et al. Cumulus cell gene expression predicts better cleavage-stage embryo or blastocyst development and pregnancy for ICSI patients. Hum Reprod. 2011;26(5):1035–51. 21372047 10.1093/humrep/der036

[14] Adriaenssens T, Van Vaerenbergh I, Van Landuyt L, Verheyen G, De Brucker M, Camus M, et al. Non-invasive cumulus cell analysis can be applied for oocyte ranking and is useful for countries with legal restrictions on embryo generation or freezing. PLoS One. 2024;19(1):e0297040. 10.1371/journal.pone.0297040 PMC10830053 38295095

[15] Adriaenssens T, Van Vaerenbergh I, Coucke W, Segers I, Verheyen G, Anckaert E, et al. Cumulus-corona gene expression analysis combined with morphological embryo scoring in single embryo transfer cycles increases live birth after fresh transfer and decreases time to pregnancy. J Assist Reprod Genet. 2019;36(3):433–43. 30627993 10.1007/s10815-018-01398-2 PMC6439096

[16] Van Vaerenbergh I, Adriaenssens T, Coucke W, Van Landuyt L, Verheyen G, De Brucker M, et al. Improved clinical outcomes after non-invasive oocyte selection and day 3 eSET in ICSI patients. Reprod Biol Endocrinol. 2021;19(1):26. 33608027 10.1186/s12958-021-00704-5 PMC7892761

[17] Adriaenssens T, Wathlet S, Segers I, Verheyen G, De Vos A, Van der Elst J, et al. Cumulus cell gene expression is associated with oocyte developmental quality and influenced by patient and treatment characteristics. Hum Reprod. 2010;25(5):1259–70. 20228394 10.1093/humrep/deq049

[18] Brannian J, Eyster K, Mueller BA, Bietz MG, Hansen K. Differential gene expression in human granulosa cells from recombinant FSH versus human menopausal gonadotropin ovarian stimulation protocols. Reprod Biol Endocrinol. 2010;8:25. 20226040 10.1186/1477-7827-8-25 PMC2842272

[19] Grøndahl ML, Borup R, Lee YB, Myrhøj V, Meinertz H, Sørensen S. Differences in gene expression of granulosa cells from women undergoing controlled ovarian hyperstimulation with either recombinant follicle-stimulating hormone or highly purified human menopausal gonadotropin. Fertil Steril. 2009;91(5):1820–30. 18439596 10.1016/j.fertnstert.2008.02.137

[20] Massoud G, Spann M, Vaught KC, Das S, Dow M, Cochran R, et al. Biomarkers assessing the role of cumulus cells on IVF outcomes: a systematic review. J Assist Reprod Genet. 2024;41(2):253–75. 37947940 10.1007/s10815-023-02984-9 PMC10894783

[21] Ferraretti AP, La Marca A, Fauser BC, Tarlatzis B, Nargund G, Gianaroli L, et al. ESHRE consensus on the definition of “poor response” to ovarian stimulation for in vitro fertilization: the Bologna criteria. Hum Reprod. 2011;26(7):1616–24. 21505041 10.1093/humrep/der092

[22] Teede HJ, Misso ML, Costello MF, Dokras A, Laven J, Moran L, et al. Recommendations from the international evidence-based guideline for the assessment and management of polycystic ovary syndrome. Hum Reprod. 2018;33(9):1602–18. 30052961 10.1093/humrep/dey256 PMC6112576

[23] Wathlet S, Adriaenssens T, Segers I, Verheyen G, Van Landuyt L, Coucke W, et al. Pregnancy prediction in single embryo transfer cycles after ICSI using QPCR: validation in oocytes from the same cohort. PLoS ONE. 2013;8(4):e54226. 23573182 10.1371/journal.pone.0054226 PMC3616108

[24] Van Landuyt L, De Vos A, Joris H, Verheyen G, Devroey P, Van Steirteghem A. Blastocyst formation in in vitro fertilization versus intracytoplasmic sperm injection cycles: influence of the fertilization procedure. Fertil Steril. 2005;83:1397–403. 15866575 10.1016/j.fertnstert.2004.10.054

[25] WHO—World Health Organization. WHO Laboratory Manual for the Examination and Processing of Human Semen, 6th edn. Geneva, Switzerland: WHO Press, 2021. https://www.who.int/publications/i/item/9789240030787

[26] Wouters K, Mateizel I, Segers I, Van de Velde H, Van Landuyt L, De Vos A, et al. Clinical pregnancy rates after blastocyst culture at a stable temperature of 36.6°C versus 37.1°C: a prospective randomized controlled trial. Hum Reprod. 2024;39(10):2233–9. 39241807 10.1093/humrep/deae193

[27] Segers I, Mateizel I, Van Moer E, Smitz J, Tournaye H, Verheyen G, et al. In vitro maturation (IVM) of oocytes recovered from ovariectomy specimens in the laboratory: a promising “ex vivo” method of oocyte cryopreservation resulting in the first report of an ongoing pregnancy in Europe. J Assist Reprod Genet. 2015;32(8):1221–31. 26253691 10.1007/s10815-015-0528-9 PMC4554385

[28] De Munck N, Santos-Ribeiro S, Mateizel I, Verheyen G. Reduced blastocyst formation in reduced culture volume. J Assist Reprod Genet. 2015;32:1365–70. 26292800 10.1007/s10815-015-0541-z PMC4595393

[29] Gardner DK, Schoolcraft WB. Culture and transfer of human blastocysts. Curr Opin Obstet Gynecol. 1999;11(3):307–11. 10.1097/00001703-199906000-00013 10369209

[30] Alpha Scientists in Reproductive Medicine and ESHRE Special Interest Group of Embryology. The Istanbul consensus workshop on embryo assessment: proceedings of an expert meeting. Hum Reprod. 2011;26(6):1270–83. 21502182 10.1093/humrep/der037

[31] Van Landuyt L, Polyzos NP, De Munck N, Blockeel C, Van de Velde H, Verheyen G. A prospective randomized controlled trial investigating the effect of artificial shrinkage (collapse) on the implantation potential of vitrified blastocysts. Hum Reprod. 2015;30:2509–18. 26364080 10.1093/humrep/dev218

[32] Canosa S, Adriaenssens T, Coucke W, Dalmasso P, Revelli A, Benedetto C, et al. Zona pellucida gene mRNA expression in human oocytes is related to oocyte maturity, zona inner layer retardance and fertilization competence. Mol Hum Reprod. 2017;23(5):292–303. 28204536 10.1093/molehr/gax008

[33] Wathlet S, Adriaenssens T, Segers I, Verheyen G, Janssens R, Coucke W, et al. New candidate genes to predict pregnancy outcome in single embryo transfer cycles when using cumulus cell gene expression. Fertil Steril. 2012;98(2):432-9.e1-4. 22633264 10.1016/j.fertnstert.2012.05.007

[34] Awwad J, Peramo B, Elgeyoushi B, Melado L, Salame A, Chawla M, et al. FSH/LH co-stimulation in advanced maternal age (AMA) and hypo-responder patients - Arabian gulf delphi consensus group. Front Endocrinol (Lausanne). 2024;15:1506332. 39726844 10.3389/fendo.2024.1506332 PMC11669953

[35] Humaidan P, Chin W, Rogoff D, D’Hooghe T, Longobardi S, Hubbard J, et al. Efficacy and safety of follitropin alfa/lutropin alfa in ART: a randomized controlled trial in poor ovarian responders. Hum Reprod. 2017;32(3):544–55 Erratum in: Hum Reprod. 2017 Jul 1;32(7):1537-1538.28137754

[36] Bosch E, Labarta E, Crespo J, Simón C, Remohí J, Pellicer A. Impact of luteinizing hormone administration on gonadotropin-releasing hormone antagonist cycles: an age-adjusted analysis. Fertil Steril. 2011;95(3):1031–6. 10.1016/j.fertnstert.2010.10.021 21067717

[37] Smitz J, Andersen AN, Devroey P, Arce JC, MERIT Group. Endocrine profile in serum and follicular fluid differs after ovarian stimulation with HP-hMG or recombinant FSH in IVF patients. Hum Reprod (Oxford, England). 2007;22(3):676–87. 10.1093/humrep/del445 17110397

[38] Egawa M, Yoshioka S, Higuchi T, Sato Y, Tatsumi K, Fujiwara H, et al. Ephrin B1 is expressed on human luteinizing granulosa cells in corpora lutea of the early luteal phase: the possible involvement of the B class Eph-ephrin system during corpus luteum formation. J Clin Endocrinol Metab. 2003;88(9):4384–92. 12970314 10.1210/jc.2002-021910

[39] Buensuceso AV, Deroo BJ. “The Ephrin signaling pathway regulates morphology and adhesion of mouse granulosa cells in vitro.” Biol Reprod. 2013;88(1):25. 23242526 10.1095/biolreprod.112.100123

[40] Ochsner SA, Day AJ, Rugg MS, Breyer RM, Gomer RH, Richards JS. Disrupted function of tumor necrosis factor-α-stimulated gene 6 blocks cumulus cell-oocyte complex expansion. Endocrinology. 2003;144:4376–84. 12959984 10.1210/en.2003-0487

[41] Fragouli E, Wells D, Iager AE, Kayisli UA, Patrizio P. Alteration of gene expression in human cumulus cells as a potential indicator of oocyte aneuploidy. Hum Reprod. 2012;27(8):2559–68. 10.1093/humrep/des170 22617123

[42] Cetica P, Pintos L, Dalvit G, Beconi M. Involvement of enzymes of amino acid metabolism and tricarboxylic acid cycle in bovine oocyte maturation in vitro. Reproduction. 2003;126(6):753–63. 14748694

[43] Humpherson PG, Leese HJ, Sturmey RG. Amino acid metabolism of the porcine blastocyst. Theriogenology. 2005;64(8):1852–66. 15923030 10.1016/j.theriogenology.2005.04.019

[44] Lamb VK, Leese HJ. Uptake of a mixture of amino acids by mouse blastocysts, (in eng). J Reprod Fertil. 1994;102(1):169–75. 7799310 10.1530/jrf.0.1020169

[45] Lee YS, Thouas GA, Gardner DK. 2015 “Developmental kinetics of cleavage stage mouse embryos are related to their subsequent carbohydrate and amino acid utilization at the blastocyst stage.” Hum Reprod. 2015;30(3):543–52. 25567621 10.1093/humrep/deu334

[46] Orsi NM, Leese HJ. Ammonium exposure and pyruvate affect the amino acid metabolism of bovine blastocysts *in vitro*. Reproduction. 2004;127(1):131–40. 15056778 10.1530/rep.1.00031

[47] Wale PL, Gardner DK. Oxygen regulates amino acid turnover and carbohydrate uptake during the preimplantation period of mouse embryo development, (in Eng). Biol Reprod. 2012;87(1):1–8. 10.1095/biolreprod.112.100552 22553221

[48] Lee YSL, Gardner DK. Early cleaving embryos result in blastocysts with increased aspartate and glucose consumption, which exhibit different metabolic gene expression that persists in placental and fetal tissues. J Assist Reprod Genet. 2021;38(12):3099–111. 34705191 10.1007/s10815-021-02341-8 PMC8666392

[49] Mitchell M, Cashman KS, Gardner DK, Thompson JG, Lane M. Disruption of mitochondrial malate-aspartate shuttle activity in mouse blastocysts impairs viability and fetal growth. Biol Reprod. 2009;80:295–301. 18971426 10.1095/biolreprod.108.069864 PMC2804819

[50] Feuerstein P, Cadoret V, Dalbies-Tran R, Guerif F, Bidault R, Royere D. Gene expression in human cumulus cells: one approach to oocyte competence. Hum Reprod. 2007;22(12):3069–77. 10.1093/humrep/dem336 17951581

[51] Faizal AM, Elias MH, Jin NM, Abu MA, Syafruddin SE, Zainuddin AA, et al. Unravelling the role of HAS2, GREM1, and PTGS2 gene expression in cumulus cells: implications for human oocyte development competency - a systematic review and integrated bioinformatic analysis. Front Endocrinol (Lausanne). 2024;15:1274376. 38524634 10.3389/fendo.2024.1274376 PMC10957552

[52] Yung Y, Ophir L, Yerushalmi GM, Baum M, Hourvitz A, Maman E. HAS2-AS1 is a novel LH/hCG target gene regulating HAS2 expression and enhancing cumulus cells migration. J Ovarian Res. 2019;12(1):21. 30819231 10.1186/s13048-019-0495-3 PMC6396505

[53] Dauphinee SM, Clayton A, Hussainkhel A, Yang C, Park YJ, Fuller ME, et al. SASH1 is a scaffold molecule in endothelial TLR4 signaling. J Immunol. 2013;191(2):892–901. 23776175 10.4049/jimmunol.1200583

[54] Christenson LK, Gunewardena S, Hong X, Spitschak M, Baufeld A, Vanselow J. Research resource: preovulatory LH surge effects on follicular theca and granulosa transcriptomes. Mol Endocrinol. 2013;27(7):1153–71. 23716604 10.1210/me.2013-1093 PMC3706842

[55] Anderson RA, Sciorio R, Kinnell H, Bayne RA, Thong KJ, de Sousa PA, et al. Cumulus gene expression as a predictor of human oocyte fertilisation, embryo development and competence to establish a pregnancy. Reproduction. 2009;138(4):629–37. 19602522 10.1530/REP-09-0144

[56] Gebhardt KM, Feil DK, Dunning KR, Lane M, Russell DL. Human cumulus cell gene expression as a biomarker of pregnancy outcome after single embryo transfer. Fertil Steril. 2011;96(1):47-52.e2. 21575950 10.1016/j.fertnstert.2011.04.033

[57] Ocampo A, Pedraza J, Ortiz G, Hernández-Pérez E, Porchia L, López-Bayghen E. Assessment of prostaglandin-endoperoxide synthase 2 and Versican gene expression profile from the cumulus cells: association with better in vitro fertilization outcomes. J Ovarian Res. 2018;11(1):84. 30241554 10.1186/s13048-018-0456-2 PMC6148785

