# Supplementary material for: Live birth in patients stimulated with r-hFSH or r-hFSH: r-hLH is strongly associated with cumulus cell derived gene expression models
Source: Reprod Biol Endocrinol. 2025 Nov 22;23:163. doi: 10.1186/s12958-025-01480-2 (PMC12751414; doi:10.1186/s12958-025-01480-2)

Supplementals

**Supplementary Table 1a:**

Spearman correlation analysis for patient characteristics in the r-hFSH stimulated group. Correlations with Spearman r >0.5 or <-0.5 and p < 0.0023 were highlighted.


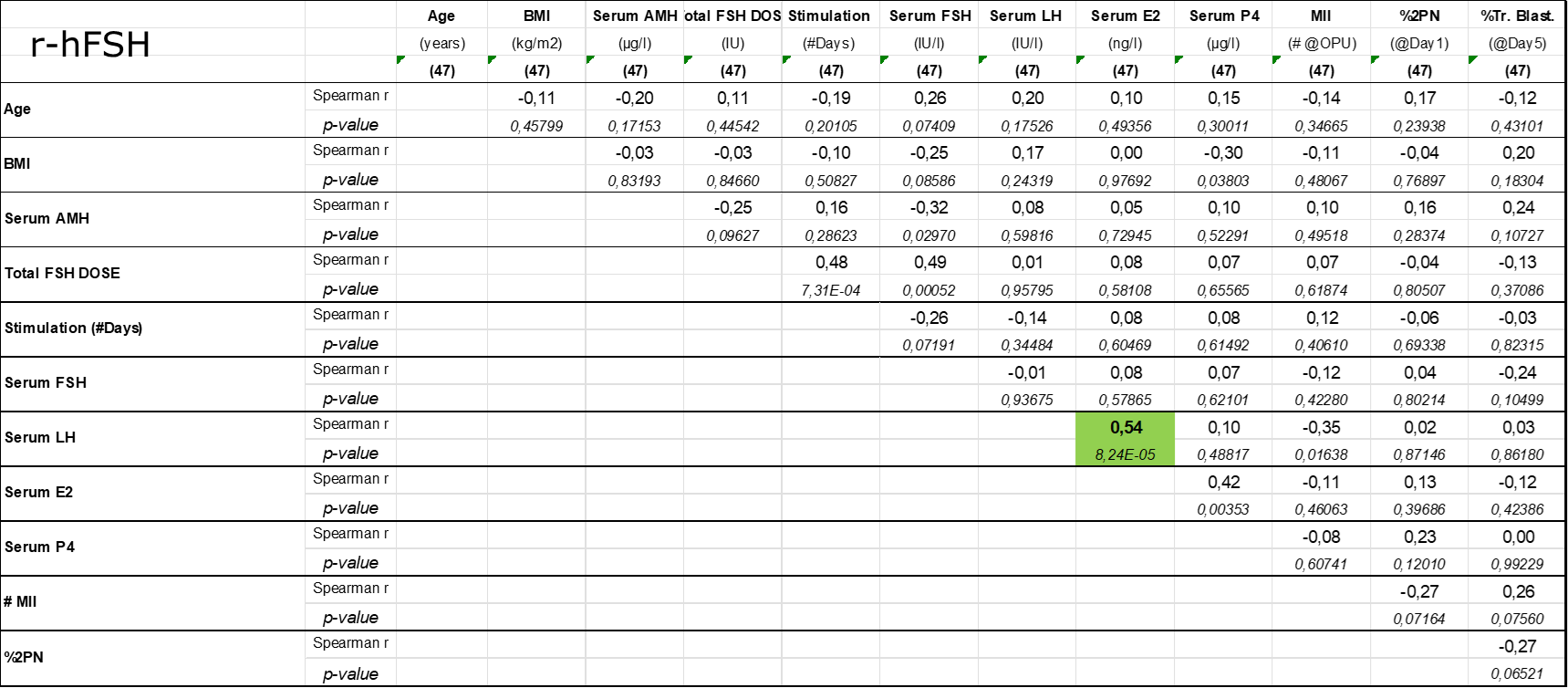


**Supplementary Table 1b:**

Spearman correlation analysis for patient characteristics in the r-hFSH: r-hLH stimulated group. Correlations with Spearman r >0.5 or <-0.5 and p < 0.0023 were highlighted.


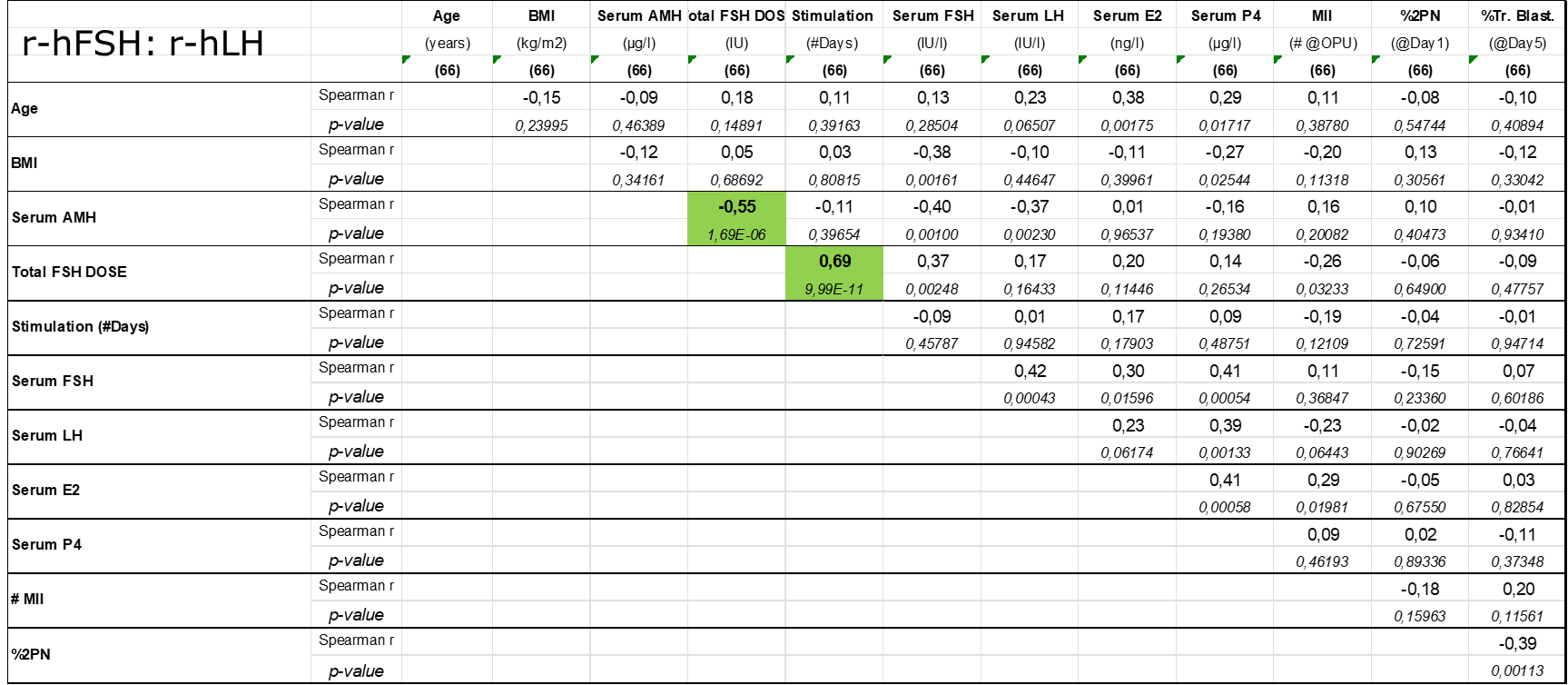


**Supplementary Table 2a and 2b:**

The analysis was repeated in a multiparametric approach (MANOVA, using R) for the largest (n=1123, Table 2a) and the smallest sample set (n=285, Table 2b). The results obtained for “CC of all oocytes (n=1123)” and “CC of all oocytes forming a GQE on day 5 (n=285)” were comparable. The expression in the two treatment groups was statistically different. This difference was detectable for EFNB2 and GOT1 but also for CAMK1D and SLC6A9. The largest differences observed were between 0.5 and 0.75. As this were log2 values actual differences were between 1.4-1.7 fold or 40% to 70% higher/lower expression between the two stimulation groups.


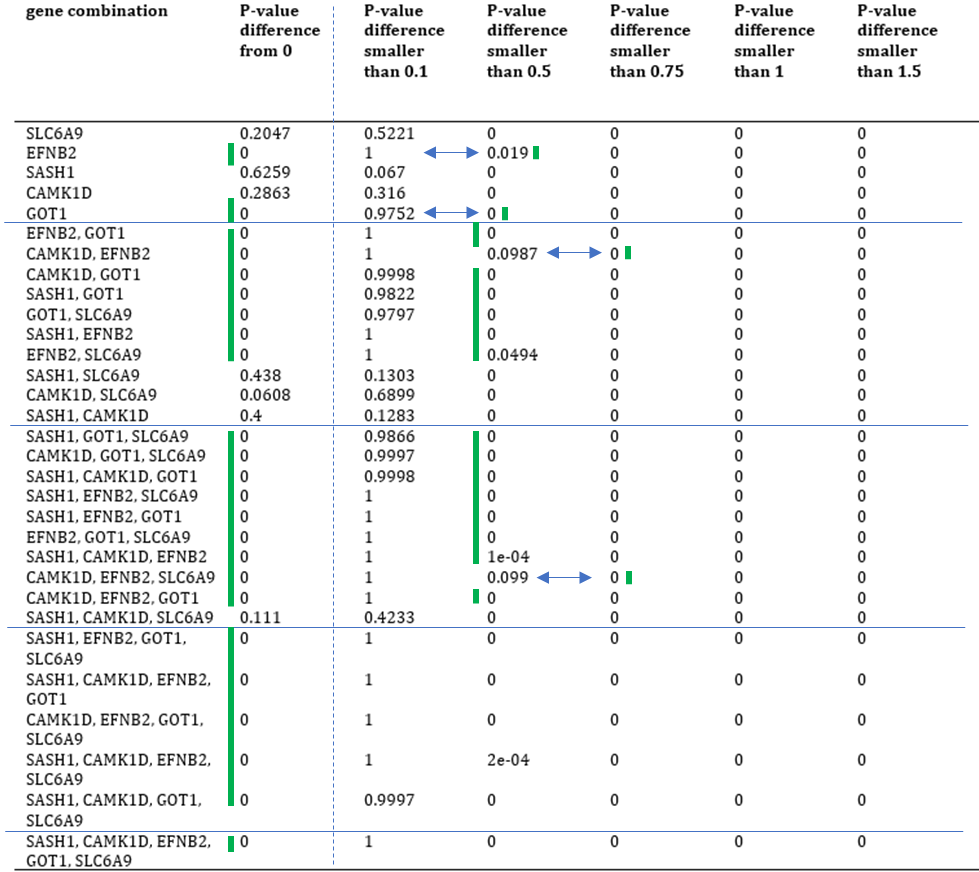


Supplementary Table 2a: MANOVA results when comparing the CC gene expression in “All oocytes” sample set. P values are enumerated in the columns for 6 hypothesis (difference between the 2 groups ><0, <0.1, <0.5, <0.75, <1, <1.5log2), and the analysis was repeated for all combinations comprising 1, 2, 3, 4 or 5 gene expressions. All analyses were performed in R. The green line (indicating significance) is added for the ease of interpretation of the table. The first column detects difference between the 2 treatment groups. If this is 0=significant then the 5 columns to the right are considered. These allow an estimation on how large the difference is between the 2 treatments. Blue arrows indicate the largest differences observed when considering the expression of only 1 or 2 or 3 genes.


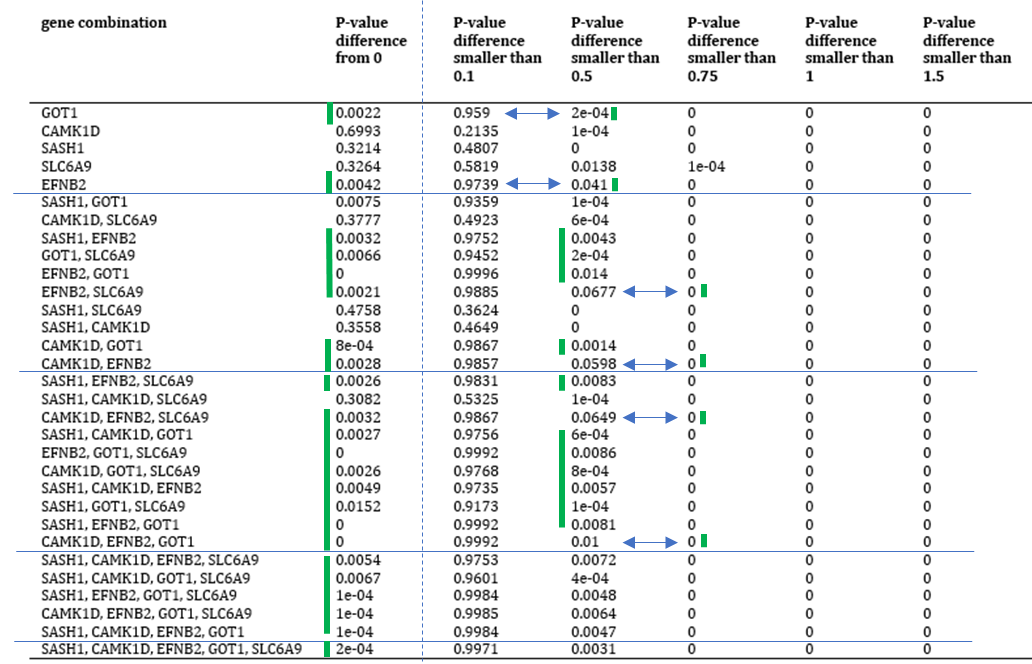


Supplementary Table 2b: MANOVA results when comparing the CC gene expression in “All oocytes forming a GQE on day 5” sample set. P values are enumerated in the columns for 6 hypothesis (difference between the 2 groups ><0, <0.1, <0.5, <0.75, <1, <1.5 log2), and the analysis was repeated for all combinations comprising 1, 2, 3, 4 or 5 gene expressions. All analyses were performed in R. The green line (indicating significance) is added for the ease of interpretation of the table. The first column detects difference between the 2 treatment groups. If this is 0=significant then the 5 columns to the right are considered. These allow an estimation on how large the difference is between the 2 treatments. Blue arrows indicate the largest differences observed when considering the expression of only 1 or 2 or 3 genes.

**Supplementary Table 3a:**

Spearman correlation analysis for patient characteristics and gene expression data in the r-hFSH stimulated group. Correlations with Spearman r >0.5 or <-0.5 and p < 0.0023 were highlighted and discussed in detail.


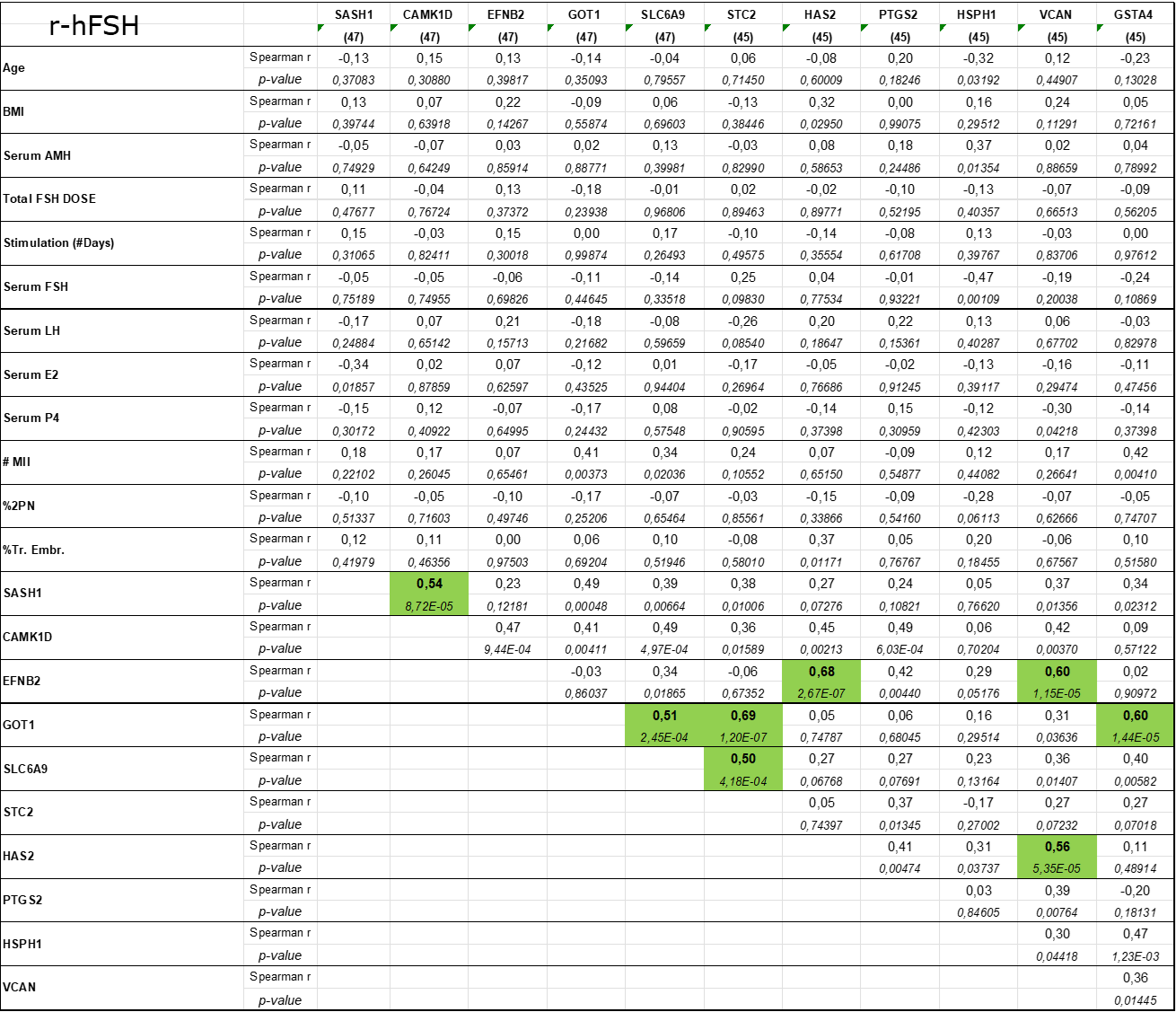


**Supplementary Table 3b:**

Spearman correlation analysis for patient characteristics and gene expression data in the r-hFSH: r-hLH stimulated group. Correlations with Spearman r >0.5 or <-0.5 and p < 0.0023 were highlighted and discussed in detail.


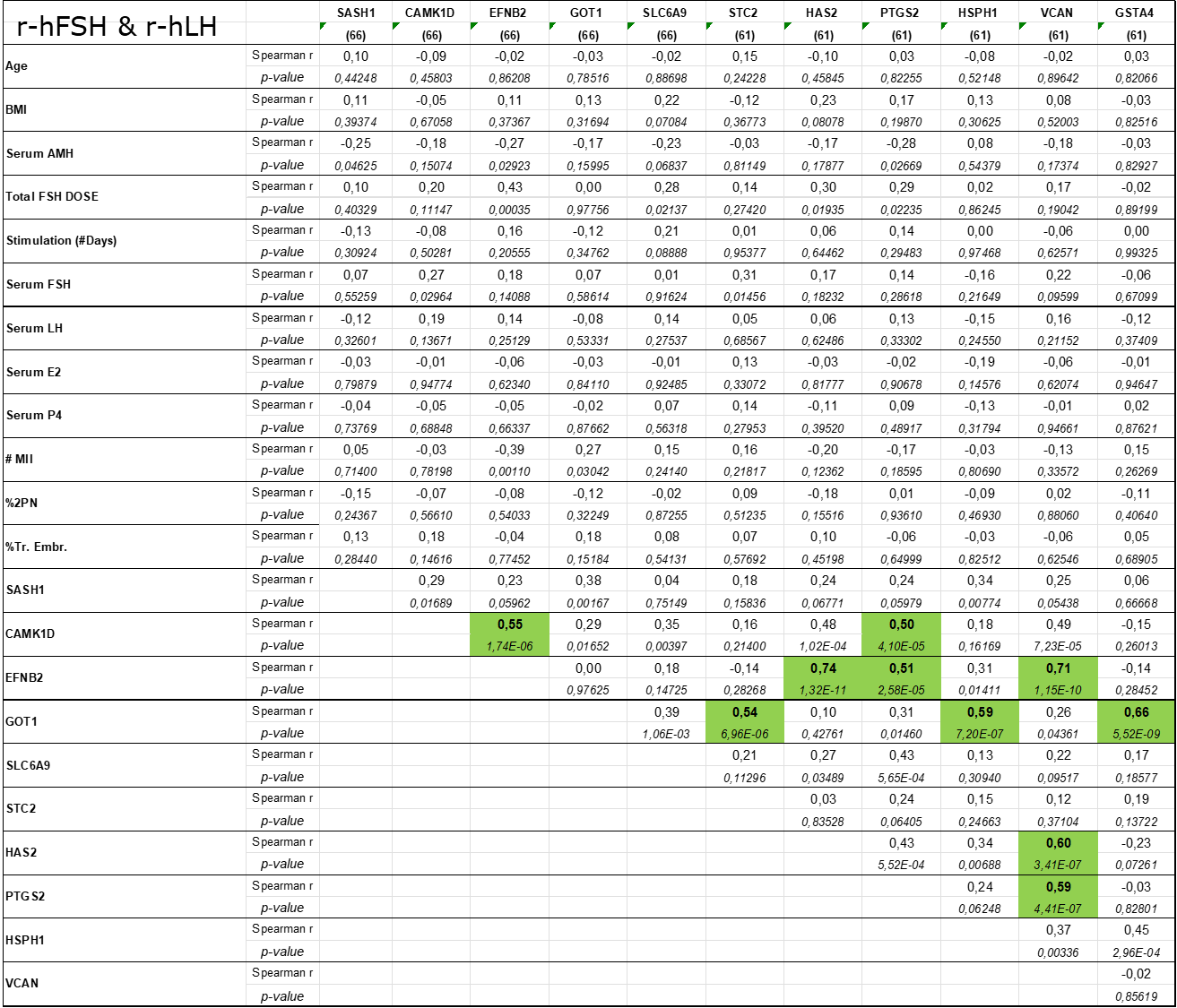

Supplement: Supplementary file 1 — Supplementary Material 1. Supplementary Table 1a: Spearman correlation analysis for patient characteristics in the r-hFSH stimulated group. Correlations with Spearman r >0.5 or <-0.5 and p < 0.0023 were highlighted. Supplementary Table 1b: Spearman correlation analysis for patient characteristics in the r-hFSH: r-hLH stimulated group. Correlations with Spearman r >0.5 or <-0.5 and p < 0.0023 were highlighted. Supplementary Table 2a and b: The analysis was repeated in a multiparametric approach (MANOVA, using R) for the largest (n=1123, Table 2a) and the smallest sample set (n=285, Table 2b). The results obtained for “CC of all oocytes (n=1123)” and “CC of all oocytes forming a GQE on day 5 (n=285)” were comparable. The expression in the two treatment groups was statistically different. This difference was detectable for EFNB2 and GOT1 but also for CAMK1D and SLC6A9. The largest differences observed were between 0.5 and 0.75. As this were log2 values actual differences were between 1.4-1.7 fold or 40% to 70% higher/lower expression between the two stimulation groups. Supplementary Table 2a: MANOVA results when comparing the CC gene expression in “All oocytes” sample set. P values are enumerated in the columns for 6 hypothesis (difference between the 2 groups ><0, <0.1, <0.5, <0.75, <1, <1.5log2), and the analysis was repeated for all combinations comprising 1, 2, 3, 4 or 5 gene expressions. All analyses were performed in R. The green line (indicating significance) is added for the ease of interpretation of the table. The first column detects difference between the 2 treatment groups. If this is 0=significant then the 5 columns to the right are considered. These allow an estimation on how large the difference is between the 2 treatments. Blue arrows indicate the largest differences observed when considering the expression of only 1 or 2 or 3 genes. Supplementary Table 2b: MANOVA results when comparing the CC gene expression in “All oocytes forming a GQE on day 5” samp [file 12958_2025_1480_MOESM1_ESM.docx]
